# DJ-1 is indispensable for the S-nitrosylation of Parkin, which maintains function of mitochondria

**DOI:** 10.1038/s41598-020-61287-6

**Published:** 2020-03-09

**Authors:** Kentaro Ozawa, Hiroki Tsumoto, Yuri Miura, Junji Yamaguchi, Sanae M. M. Iguchi-Ariga, Tetsushi Sakuma, Takashi Yamamoto, Yasuo Uchiyama

**Affiliations:** 1 Research Team for Mechanism of Aging, Tokyo Metropolitan Institute of Gerontology, 35-2 Sakae-cho, Itabashi-ku, Tokyo 173-0015 Japan; 2 Department of Pharmacology, Nara Medical University School of Medicine, Kashihara City, Nara 634-8521 Japan; 3 Department of Cellular and Molecular Neuropathology, Juntendo University Graduate School of Medicine, Bunkyo-Ku 2-1-1, Tokyo, 113-8421 Japan; 4 Asakayama General Hospital, Sakai-ku, Sakai City, Osaka 590-0018 Japan; 5 Faculty of Pharmaceutical Sciences, Hokkaido University, Kita 12, Nishi 6, Kita-ku, Sapporo 060-0812 Japan; 6 Division of Integrated Sciences for Life, Graduate School of Integrated Sciences for Life, Hiroshima University, Hiroshima, 739-8526 Japan

**Keywords:** Neurological disorders, Cell biology, Mechanisms of disease, Biochemistry, Proteomics, Neuroscience, Diseases of the nervous system

## Abstract

The DJ-1 gene, a causative gene for familial Parkinson’s disease (PD), has been reported to have various functions, including transcriptional regulation, antioxidant response, and chaperone and protease functions; however, the molecular mechanism associated with the pathogenesis of PD remains elusive. To further explore the molecular function of DJ-1 in the pathogenesis of PD, we compared protein expression profiles in brain tissues from wild-type and DJ-1-deficient mice. Two-dimensional difference gel electrophoresis analysis and subsequent analysis using data mining methods revealed alterations in the expression of molecules associated with energy production. We demonstrated that DJ-1 deletion inhibited S-nitrosylation of endogenous Parkin as well as overexpressed Parkin in neuroblastoma cells and mouse brain tissues. Thus, we used genome editing to generate neuroblastoma cells with DJ-1 deletion or S-nitrosylated cysteine mutation in Parkin and demonstrated that these cells exhibited similar phenotypes characterized by enhancement of cell death under mitochondrial depolarization and dysfunction of mitochondria. Our data indicate that DJ-1 is required for the S-nitrosylation of Parkin, which positively affects mitochondrial function, and suggest that the denitrosylation of Parkin via DJ-1 inactivation might contribute to PD pathogenesis and act as a therapeutic target.

## Introduction

The protein deglycase DJ-1 is encoded by the PARK7 gene in humans^1,2^. The DJ-1 gene was originally identified as an oncogene that enhances the Ras/MAPK pathway and transforms fibroblastic cells^1^ and was later identified as a causative gene for autosomal recessive juvenile parkinsonism (ARJP)^1,2^. DJ-1 has been reported to play important roles in various cellular functions, including antioxidant response mediation and mitochondrial regulation^1^. The Cys106 residue of DJ-1 is highly susceptible to oxidative stress and is oxidized, and its mutation results in loss of DJ-1 activity^3^. DJ-1 has been reported to exert neuroprotective effects at least in part via antioxidant defense; however, the mechanisms by which DJ-1 protects neurons from oxidative stress and mutation of the DJ-1 gene contributes to the pathogenesis of PD remain to be elucidated.

Studies in animal and cellular models have verified that DJ-1, Parkin and pten-induced kinase 1 (PINK1) are linked. In Drosophila, deletion of parkin and pink1, which are also causative genes for ARJP, causes morphological transformation and mitochondrial dysfunction in energy-demanding tissues including muscles and brain^4^. Upregulation of parkin rescues the effects of pink1 mutation, thus indicating that these two genes are in the same genetic pathway and that parkin is downstream of pink1. Flies with dj-1 deletion exhibit similar biological phenotypes as flies with either pink1 or parkin deletion, and upregulation of fly dj-1 or human DJ-1 rescues pink1 but not parkin deficiency^5^. In primary neurons and transformed cells, overexpression of either Parkin or PINK1 rescues the mitochondrial fragmentation caused by the loss of DJ-1^6,7^. Overexpression of DJ-1 protects against mitochondrial dysfunction in cells transiently transfected with shRNA targeting PINK1 and rescues the vulnerability of dopaminergic neurons to an inhibitor of mitochondrial complex I in mice with PINK1 deletion^8^. Collectively, these findings indicate that DJ-1 operates parallel to PINK1, but not Parkin, in a pathway to maintain mitochondrial function during exposure to stress.

Under steady-state conditions, PINK1 is continuously cleaved and degraded in a ubiquitin-dependent manner^9,10^. Upon mitochondrial depolarization, PINK1 is expressed and activated on the outer mitochondrial membrane (OMM), and thus stabilized PINK1 phosphorylates both ubiquitin and Parkin at their respective Ser65 residues. Phosphorylated ubiquitin binds with high affinity to phosphorylated Parkin, leading to induction of conformational changes that increase the E3 ligase activity of Parkin. PINK1-Parkin-dependent signaling initiates ubiquitination of proteins on the OMM, resulting in recruitment of the autophagy machinery. Despite the overwhelming evidence for this interaction, few studies have been able to identify a link between DJ-1 and Parkin or PINK1.

In this study, to analyze the expression of proteins in brain tissues from wild-type (WT) and DJ-1-deficient mice, we used a two-dimensional fluorescence difference gel electrophoresis (2D-DIGE) technique to generate quantitative protein expression profiles, which indicated that DJ-1 deletion changed the expression of proteins involved in energy production. In addition, we revealed that DJ-1 deletion inhibited the S-nitrosylation of endogenous Parkin in neuroblastoma cells and mouse brain tissues. Thus, we used genome editing to generate neuroblastoma cells with DJ-1 deletion and an S-nitrosylated cysteine mutation in Parkin and showed that these two mutated cell lines showed similar mitochondrial functional phenotypes.

## Results

### 2D-DIGE analysis of brain tissues from DJ-1-deficient mice

To study the role of DJ-1 in neurodegeneration, proteomic analysis of brain tissues from DJ-1-deficient mice (DJ-1^−/−^ mice) and age-matched C57BL/6 (wild-type) mice was performed using 2D-DIGE. For our experiments, we routinely prepared protein samples from three individual four- to eight-week-old male wild-type and DJ-1^−/−^ mice. A representative 2D gel image is shown in Supplemental Fig. S1. On each gel, 223 spots were detected by spot analysis. Sixteen of these spots represented proteins with increased expression (marked with red circles) in brain tissues from DJ-1^−/−^ mice, and 17 represented proteins with decreased expression (blue circles) in brain tissues from DJ-1^−/−^ mice. Mass spectrometric analysis identified 87 proteins, 33 of which were from spots with signals that were changed in brain tissues from DJ-1^−/−^ mice, as listed in Supplemental Table S1.

**Figure 1:**
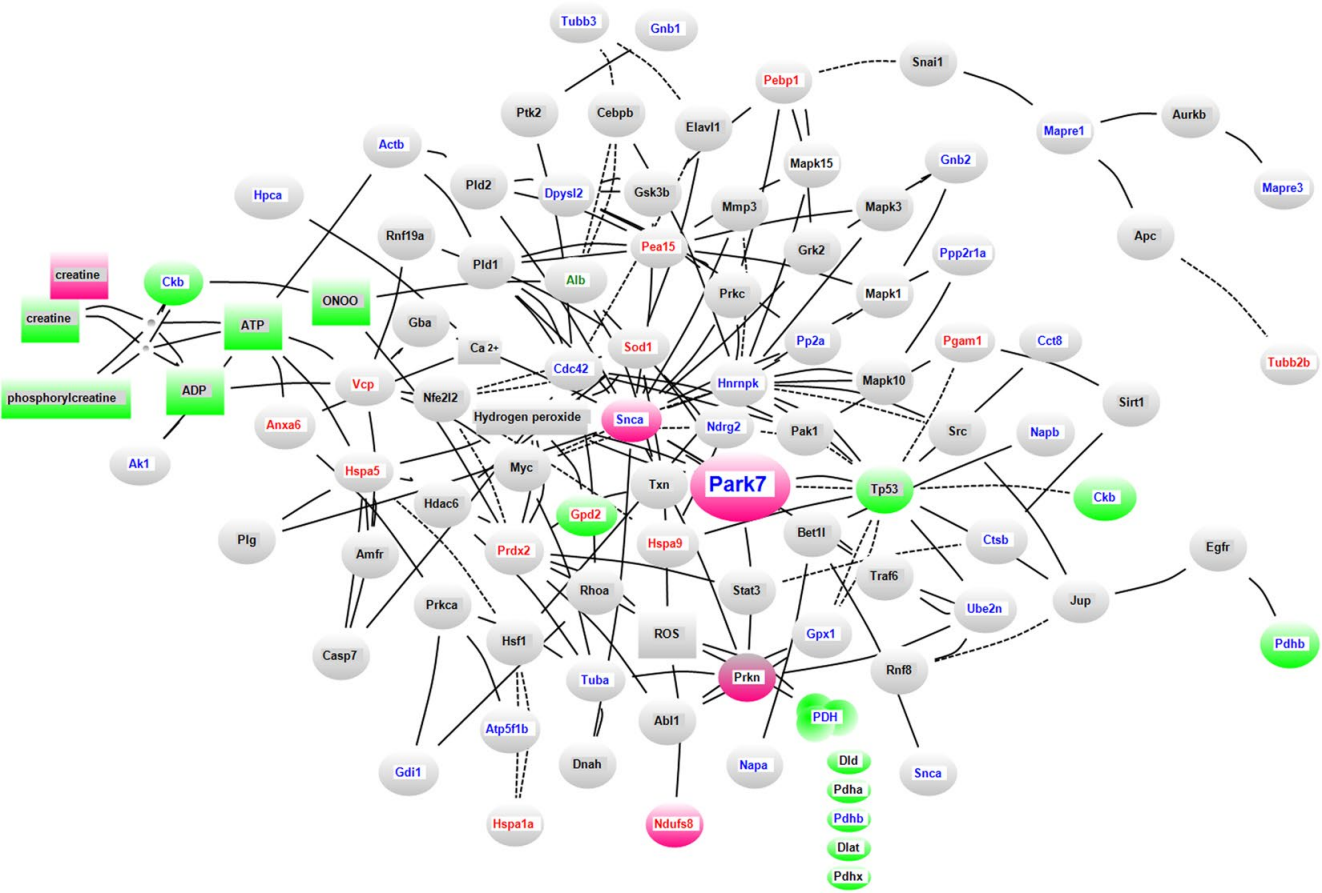
Molecular network identified using the “Interaction search” algorithm in KeyMolnet. Molecules with terms in red, blue, or green text represent proteins with increased, decreased, or both increased and decreased spots, respectively, in samples from DJ-1^−/−^ mice compared with samples from wild-type mice. The terms in black text represent molecules related to differentially expressed proteins in DJ-1^−/−^ mice based on the “Interaction search” algorithm in KeyMolnet. The green background represents molecules related to the glycolytic pathway, creatine pathway, and TCA cycle. The pink background represents molecules related to PD. The gray background represents other molecules. Molecules surrounded by an ellipse, triangle, or square represent proteins, protein complexes, or small molecules, respectively. The solid and dotted lines represent active and inactive interactions, respectively, between end molecules. The protein abbreviations represent the gene names summarized in Supplemental Table S1, and those of other molecules correspond to those in KeyMolnet.

The proteomic analysis data were subjected to gene ontology analysis using the Protein Analysis Through Evolutionary Relationships (PANTHER) classification system^11^, which showed that the differentially expressed proteins in brain tissues from DJ-1^−/−^ mice were mainly associated with the “binding” and “catalytic activity” rather than the “antioxidant activity” molecular function categories, the “cellular process” and “metabolic process” biological process categories, and the “hydrolase” protein class category (Supplemental Fig. S2). Furthermore, we employed pathway analysis using the Kyoto Encyclopedia of Genes and Genomes (KEGG)^12^ and KeyMolnet^13^ platforms to extract the molecular networks biologically relevant to the differentially expressed proteins in brain tissues from DJ-1^−/−^ mice. KEGG analysis extracted “phagosome” as a relevant pathway enriched in differentially expressed proteins in brain tissues from DJ-1^−/−^ mice, suggesting that DJ-1 might be involved in lysosomal degradation (Supplemental Table S2). KeyMolnet analysis revealed highly relevant functional networks closely associated with aspects of energy production (green molecules in Fig. 1), such as glycolysis (score 41.9), creatine pathway (score 38.0), tricarboxylic acid (TCA) cycle (score 34.3), ROS signaling pathway (score 40.6), and autophagy-related protein signaling pathway (score 35.3), as listed in Supplemental Table S3. Additionally, mitochondrial dynamics were extracted as highly relevant pathological events (score 31.6) by KeyMolnet (Supplemental Table S3). Supplemental Table S3 summarizes the molecules associated with the highly relevant networks and pathological events extracted by KeyMolnet. Taken together, the proteomic analysis results revealed that DJ-1 deletion changed the expression of proteins related to energy production by glycolysis, the creatine pathway, and the mitochondrial TCA cycle and related to processes involving lysosomal degradation, including autophagy, suggesting that DJ-1 might be involved in the maintenance of mitochondrial function by lysosomal degradation.

### Deletion of DJ-1 causes mitochondrial dysfunction

**Figure 2:**
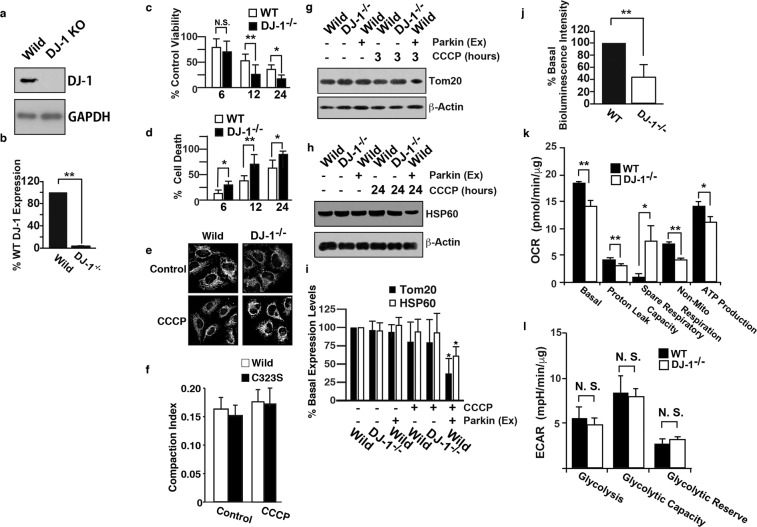
Deletion of DJ-1 suppresses mitochondrial function and cell viability via the depolarization of the mitochondrial membrane potential. (**a**) Lysates prepared from SH-SY5Y cells (WT) and DJ-1^−/−^ cells were immunoblotted with anti-DJ-1 (a, upper panel) and anti-GAPDH (a, lower panel) antibodies. (**b**) The level of DJ-1, as measured by scanning densitometry, is expressed as the percentage of that in SH-SY5Y cells (WT) normalized with respect to GAPDH. The data are shown as the means ± standard errors (SEs) (n = 3); **p < 0.01 versus SH-SY5Y cells. Cell viability (**c**) and cell death (**d**) were evaluated using trypan blue dye and by measuring LDH release, respectively. Cell viability and cell death are expressed as percentages of the control values. The data are shown as the means ± SEs (n = 6); *p < 0.05 and **p < 0.01 versus SH-SY5Y cells (WT); N.S. indicates no significant difference; ANOVA with Tukey’s honest significant difference (HSD) test. Wild-type SH-SY5Y (WT) and DJ-1^−/−^ cells were incubated with or without CCCP (10 µM) for 1 h and were then immunostained with an anti-Tom20 antibody (**e**). The compaction index was calculated from images of cells stained with an anti-Tom20 antibody, as described in the Materials and Methods section (**f**). Lysates prepared from SH-SY5Y cells (WT) and DJ-1^−/−^ cells treated with CCCP (10 µM) for different time periods as indicated were immunoblotted with anti-Tom20 (**g**), anti-HSP60 (**h**) and anti-β-actin (lower panels) antibodies. Parkin (Ex) indicates samples from SH-SY5Y cells transfected with the FLAG-tagged wild-type Parkin plasmid. (**i**) The levels of Tom20 (**g**) and HSP60 (**h**), as measured by scanning densitometry, are expressed as percentages of the levels in CCCP-untreated samples normalized with respect to β-actin (lower panels). The data are shown as the means ± SEs (n = 4); *p < 0.05 versus CCCP-untreated samples. (**j**) Evaluation of ATP in wild-type (WT) and DJ-1^−/−^ cells by a luciferase assay. The data are shown as the means ± SEs (n = 8); **p < 0.01 versus wild-type cell samples. (**k**,**l**) Measurement of the cellular oxygen consumption rate (OCR, **k**) and extracellular acidification rate (ECAR, **l**) in wild-type (WT) and DJ-1^−/−^ cells using the XFp extracellular flux analyzer, as described in the Materials and Methods section. The data are shown as the means ± standard deviations (n = 3); **p < 0.01, *p < 0.05 versus WT cells; N.S. indicates no significant difference; Student’s t-test. The data were normalized to the amount of total protein. Full scans of the blots in a, g and h are available in Supplemental Fig. S8.

Although mutations in DJ-1 as well as Parkin and PINK1 have been reported to cause ARJP^2^, it is controversial whether DJ-1 maintains mitochondrial function or protects neurons from oxidative stress caused by mitochondrial dysfunction. Additionally, the differentially expressed proteins in DJ-1-deficient brains are related to both the maintenance of mitochondrial function and the ROS signaling pathway. To elucidate the role of DJ-1 in mitochondrial homeostasis, we deleted the DJ-1 gene in SH-SY5Y cells, which are often used in PD research, by homologous recombination using transcription activator-like effector nuclease (TALEN)-mediated genome editing (Supplemental Fig. S3)^14^. The immunoblot results showed that the expression of DJ-1 was abolished in DJ-1-deficient SH-SY5Y cells (DJ-1^−/−^ cells, Fig. 2a,b). We evaluated the viability and death of wild-type and DJ-1^−/−^ cells in the presence of carbonyl cyanide m-chlorophenyl hydrazone (CCCP), which causes depolarization of the mitochondrial membrane potential, and found that cell viability was decreased and cell death was increased significantly in DJ-1^−/−^ cells compared with wild-type cells (Fig. 2c,d).

We used wild-type and DJ-1^−/−^ cells to evaluate the effect of DJ-1 on relative mitochondrial aggregation, which is induced by Parkin soon after mitochondrial depolarization, and on mitochondrial degradation. Immunostaining experiments and subsequent calculation of the compaction index^15^ showed no significant differences in mitochondrial aggregation between wild-type and DJ-1^−/−^ cells (Fig. 2e,f). In addition, the immunoblots showed degradation of Tom20 and HSP60, which are located in the OMM and matrix, respectively, in the presence of CCCP in wild-type cells with Parkin overexpression but not in wild-type or DJ-1^−/−^ cells without Parkin overexpression (Fig. 2g–i). These data indicate that, consistent with a previous study^16^, the exogenous expression of Parkin is necessary for robust mitochondrial degradation throughout the cell in the presence of CCCP in SH-SY5Y cells. We evaluated the mitochondrial membrane potential using tetramethylrhodamine methyl ester (TMRM)^17^ and 5,5′,6,6′-tetrachloro-1,1′,3,3′-tetraethylbenzimidazolylcarbocyanine iodide (JC-1)^18^ and the production of mitochondrial superoxides using MitoSOX^19^. There was no significant difference in the mitochondrial membrane potential or superoxide production between wild-type and DJ-1^−/−^ cells under normal conditions or with CCCP/rotenone (Supplemental Fig. S4), indicating that DJ-1 deletion does not affect the mitochondrial membrane potential or production of mitochondrial superoxides in neuroblastoma cells.

Then, we evaluated the ATP content in wild-type SH-SY5Y and DJ-1^−/−^ cells using bioluminescence^20^. The ATP content was significantly lower in DJ-1^−/−^ cells than in wild-type cells (Fig. 2j). Furthermore, we evaluated mitochondrial respiration and glycolytic function by measuring the cellular oxygen consumption rate (OCR) and extracellular acidification rate (ECAR) of cells, as described in the Materials and Methods section^21^. There were significant differences in the “basal OCR”, “proton leak”, “spare respiratory capacity”, “non-mito respiration” and “ATP production” aspects of mitochondrial respiration (Fig. 2k) but not in glycolytic function (Fig. 2l). Taken together, these data indicate that DJ-1 deletion affects ATP production but not mitochondrial superoxide production in SH-SY5Y cells.

### Deletion of DJ-1 denitrosylates parkin

**Figure 3:**
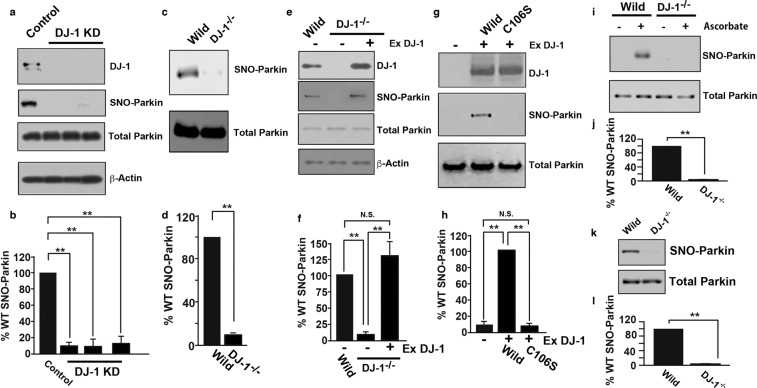
Deletion of DJ-1 denitrosylates Parkin. (**a**) Wild-type SH-SY5Y cells overexpressing FLAG-tagged wild-type Parkin were transfected with the FLAG-tagged wild-type Parkin plasmid and with a predesigned siRNA targeting DJ-1 (DJ-1 KD) or scrambled siRNA (control) and were then incubated for 48 h. Lysates were used for immunoblot analysis with anti-DJ-1, anti-FLAG (total Parkin) and anti-β-actin antibodies and SNO-RAC with subsequent immunoblot analysis with an anti-FLAG antibody (SNO-Parkin), through which ascorbate-dependent purification demonstrated the presence of S-nitrosylated cysteine residues, as indicated. (**b**) The level of S-nitrosylated Parkin, as measured by scanning densitometry, is expressed as a percentage of the control normalized with respect to total Parkin. The data are shown as the means ± SEs (n = 4); **p < 0.01 versus control. (**c**) Lysates from SH-SY5Y (WT) or DJ-1^−/−^ cells transfected with the FLAG-tagged wild-type Parkin plasmid were used for immunoblot analysis with an anti-FLAG (total Parkin) antibody and SNO-RAC with subsequent immunoblot analysis with an anti-FLAG antibody (SNO-Parkin), as indicated. (**d**) The level of S-nitrosylated Parkin, as measured by scanning densitometry, is expressed as a percentage of the control normalized with respect to total Parkin. The data are shown as the means ± SEs (n = 4); **p < 0.01 versus control. (**e**) Lysates from SH-SY5Y or DJ-1^−/−^ cells transfected with the FLAG-tagged wild-type Parkin and DJ-1 (Ex DJ-1) plasmids were used for immunoblot analysis with anti-DJ-1, anti-FLAG (Total Parkin) and anti-β-actin antibodies and SNO-RAC with subsequent immunoblot analysis with an anti-FLAG antibody (SNO-Parkin), as indicated. (**f**) The level of S-nitrosylated Parkin, as measured by scanning densitometry, is expressed as a percentage of wild-type normalized with respect to total Parkin. The data are shown as the means ± SEs (n = 4); **p < 0.01; N.S., no significant difference. (**g**) Lysates from DJ-1^−/−^ cells transfected with the FLAG-tagged Parkin and DJ-1 plasmids were used for immunoblot analysis with anti-DJ-1 and anti-FLAG (total Parkin) antibodies and SNO-RAC with subsequent immunoblot analysis with an anti-FLAG antibody (SNO-Parkin), as indicated. WT and C106S indicate transfection of wild-type and C106S DJ-1, respectively. (**h**) The level of S-nitrosylated Parkin, as measured by scanning densitometry, is expressed as a percentage of that in cells transfected with wild-type DJ-1 (WT) normalized with respect to total Parkin. The data are shown as the means ± SEs (n = 4); **p < 0.01; N.S., no significant difference. (**i**) Lysates from SH-SY5Y (WT) or DJ-1^−/−^ cells were used for immunoblot analysis with an anti-Parkin antibody (total Parkin) and SNO-RAC with subsequent immunoblot analysis with an anti-Parkin antibody (SNO-Parkin), as indicated. (**j**) The level of S-nitrosylated Parkin, as measured by scanning densitometry, is expressed as a percentage of wild-type normalized with respect to total Parkin. The data are shown as the means ± SEs (n = 4); **p < 0.01 versus wild-type cells. (**k**) Brain tissue extracts from wild-type (WT) or DJ-1^−/−^ mice were used for immunoblot analysis with an anti-Parkin antibody (total Parkin) and SNO-RAC with subsequent immunoblot analysis with an anti-Parkin antibody (SNO-Parkin), as indicated. (**l**) The level of S-nitrosylated Parkin, as measured by scanning densitometry, is expressed as a percentage of wild-type normalized with respect to total Parkin. The data are shown as the means ± SEs (n = 3); **p < 0.01 versus wild-type mice. Full scans of the blots in a, c, e, g, i and k are available in Supplemental Fig. S8.

Previously, we revealed that the S-nitrosylation of Parkin regulates its E3 ligase activity, which plays an important role in autophagic mitochondrial degradation to maintain mitochondrial homeostasis^22^. We thus hypothesized that DJ-1 might maintain mitochondrial function through the S-nitrosylation of Parkin. To reveal the effect of DJ-1 on the S-nitrosylation of Parkin, three predesigned siRNAs targeting DJ-1 and a FLAG-tagged wild-type Parkin overexpression plasmid were transfected into SH-SY5Y cells, and the S-nitrosylation of Parkin was evaluated with a modified biotin switch assay for protein S-nitrosothiols (SNOs) using resin-assisted capture (SNO-RAC)^23^. The siRNAs targeting DJ-1 almost completely eliminated the expression of DJ-1 but not Parkin, and overexpressed Parkin showed no detectable level of S-nitrosylation (Fig. 3a,b). To confirm the effect of DJ-1 on Parkin S-nitrosylation, we overexpressed Parkin in wild-type SH-SY5Y and DJ-1^−/−^ cells and evaluated Parkin S-nitrosylation. Parkin S-nitrosylation was impaired in DJ-1^−/−^ cells compared to wild-type cells (Fig. 3c,d), whereas overexpression of wild-type DJ-1 recovered Parkin S-nitrosylation in DJ-1^−/−^ cells but not in cells expressing C106S mutant DJ-1, which was reported to be an inactive mutant (Fig. 3e–h). C106S mutant DJ-1 and wild-type DJ-1 were S-nitrosylated (Supplemental Fig. S5). Furthermore, the S-nitrosylation of endogenous Parkin was impaired in both DJ-1^−/−^ cells and DJ-1^−/−^ mice (Fig. 3i–l). These data indicate that DJ-1 plays an indispensable role in the S-nitrosylation of Parkin.

### Mutation of the Cys323 residue of Parkin causes mitochondrial dysfunction

**Figure 4:**
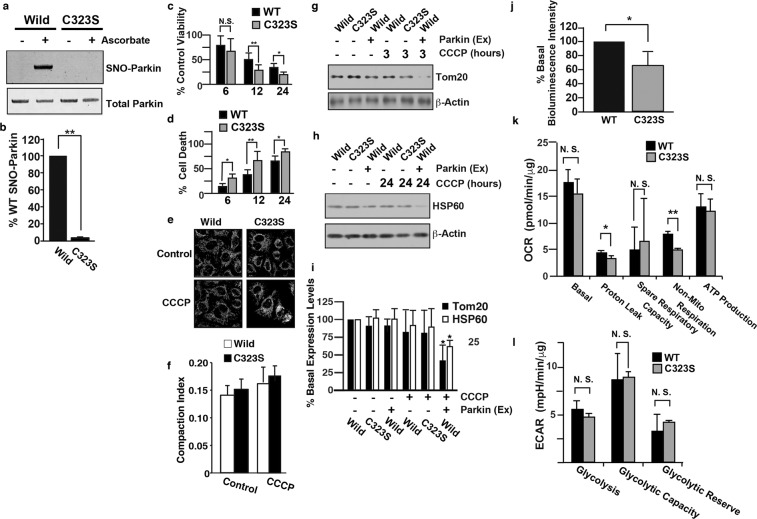
Substitution of Cys323 in Parkin suppresses mitochondrial function and cell viability via the depolarization of the mitochondrial membrane potential. (**a**) Lysates from wild-type SH-SY5Y (WT) or Parkin^C323S^ (C323S) cells were used for immunoblot analysis with an anti-Parkin antibody (total Parkin) and SNO-RAC with subsequent immunoblot analysis with an anti-Parkin antibody (SNO-Parkin), as indicated. (**b**) The level of S-nitrosylated Parkin, as measured by scanning densitometry, is expressed as a percentage of wild-type normalized with respect to total Parkin. The data are shown as the means ± SEs (n = 4); **p < 0.01 versus wild-type cells. Cell viability (**c**) and cell death (**d**) were evaluated using trypan blue dye and by measuring LDH release, respectively. Cell viability and cell death are expressed as percentages of the control values. The data are shown as the means ± SEs (n = 6); *p < 0.05 and **p < 0.01 versus control; N.S., no significant difference; ANOVA with Tukey’s HSD test. Wild-type SH-SY5Y (WT) and Parkin^C323S^(C323S) cells were incubated with or without CCCP (10 µM) for 1 h and were then immunostained with an anti-Tom20 antibody (**e**). The compaction index was calculated from images of cells stained with an anti-Tom20 antibody, as described in the Materials and Methods section (**f**). Lysates prepared from SH-SY5Y cells and Parkin^C323S^ cells treated with CCCP (10 µM) for different time periods as indicated were immunoblotted with anti-Tom20 (**g**), anti-HSP60 (**h**) and anti-β-actin (lower panels) antibodies. Parkin (Ex) indicates samples from SH-SY5Y cells transfected with the FLAG-tagged wild-type Parkin plasmid. (**i**) The levels of Tom20 (**g**) and HSP60 (**h**), as measured by scanning densitometry, are expressed as percentages of the control normalized with respect to β-actin (lower panels). The data are shown as the means ± SEs (n = 4); **p < 0.01 versus CCCP-untreated samples. (**j**) Evaluation of ATP in wild-type (WT) and Parkin^C323S^ cells by a luciferase assay. The data are shown as the means ± SEs (n = 8); **p < 0.01 versus wild-type cell samples. Measurement of the cellular oxygen consumption rate (OCR, **k**) and extracellular acidification rate (ECAR, **l**) in wild-type (WT) and Parkin^C323S^(C323S) cells using the XFp extracellular flux analyzer, as described in the Materials and Methods section. The data are shown as the means ± standard deviations (n = 3); **p < 0.01, *p < 0.05 versus WT cells; N.S., no significant difference; Student’s t-test. The data were normalized to the amount of total protein. Full scans of the blots in a, g and h are available in Supplemental Fig. S8.

We revealed that the S-nitrosylation of overexpressed Parkin activates its E3 ligase activity and mitochondrial degradation triggered by depolarization of the mitochondrial membrane potential^22^; however, the role of the S-nitrosylation of endogenous Parkin remains to be investigated. To investigate the S-nitrosylation of endogenous Parkin, we generated a construct to convert the Cys323 residue of Parkin, which is the S-nitrosylated cysteine residue, to a serine and generated mutant SH-SY5Y cells (Parkin^C323S^ cells) via homologous recombination using TALEN-mediated genome editing (Supplemental Fig. S6)^14^. The expression of Parkin in Parkin^C323S^ cells was comparable to that in wild-type SH-SY5Y cells (Supplemental Fig. S6d). Consistent with our previous report, S-nitrosylation of endogenous Parkin in wild-type cells but not in Parkin^C323S^ cells was detected by SNO-RAC (Fig. 4a,b), indicating that Cys323 is the S-nitrosylated cysteine of endogenous Parkin.

We evaluated the viability and death of wild-type and Parkin^C323S^ cells in the presence of CCCP and found that compared with that of wild-type cells the viability of Parkin^C323S^ cells was decreased and the death of Parkin^C323S^ cells was increased (Fig. 4c,d). Immunostaining and immunoblotting experiments showed no significant differences in mitochondrial aggregation (Fig. 4e,f) or the degradation of Tom20 and HSP60 (Fig. 4g–i), respectively, between wild-type and Parkin^C323S^ cells. In addition, flow cytometry showed no significant differences in the mitochondrial membrane potential or superoxide production between wild-type cells and Parkin^C323S^ cells (Supplemental Fig. S7).

The ATP content in Parkin^C323S^ cells, as measured using bioluminescence^20^, was significantly lower than that in wild-type cells (Fig. 4j). In addition, measurement of the cellular OCR and ECAR showed significant differences in the “proton leak” and “non-mito respiration” aspects of mitochondrial respiration (Fig. 4k) between wild-type and Parkin^C323S^ cells but no significant difference in glycolytic function (Fig. 4l). Taken together, these results indicate that Parkin^C323S^ cells show mitochondrial dysfunction phenotypes similar to those of DJ-1^−/−^ cells in terms of the impairment of ATP production and cell death, suggesting that DJ-1 might play an important role in the maintenance of energy production in mitochondria through the S-nitrosylation of the Cys323 residue of Parkin.

## Discussion

**Figure 5:**
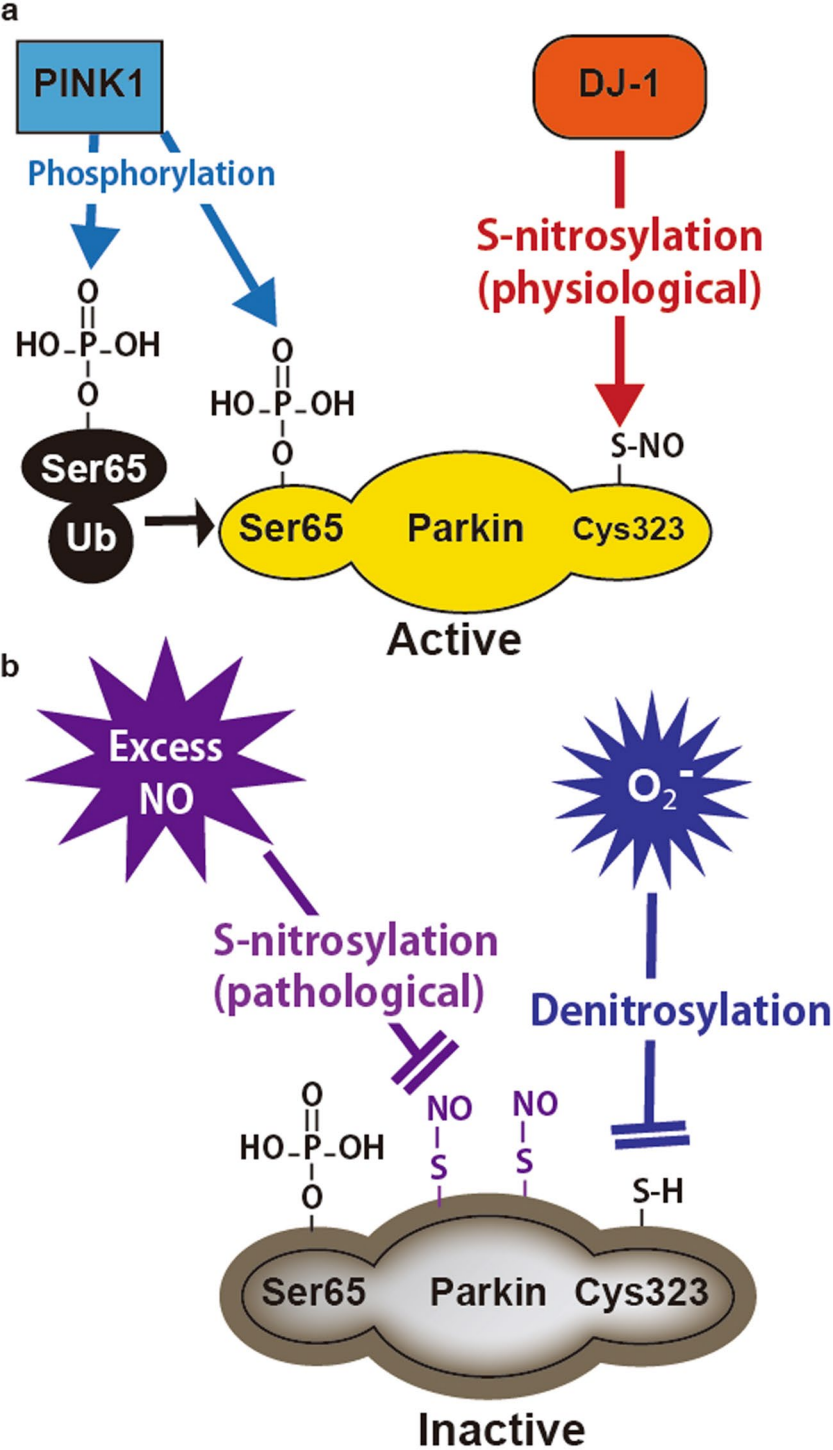
Working hypothesis of the regulation of mitochondrial function by the S-nitrosylation of Parkin. (**a**) DJ-1 physiologically S-nitrosylates the Cys323 residue of Parkin independent of the PINK1-dependent phosphorylation of Parkin and ubiquitination. The physiological S-nitrosylation of the Cys323 residue of Parkin maintains the function of mitochondria and protects neurons from cell death caused by mitochondrial dysfunction. (**b**) Excess NO may cause the pathological S-nitrosylation of one or more cysteines of Parkin other than Cys323, leading to the inactivation of Parkin. Superoxides decrease the S-nitrosylation of the Cys323 residue of Parkin (denitrosylation), which also inactivates the E3 ligase activity of Parkin.

In this report, we revealed that DJ-1 deletion inhibits the S-nitrosylation of endogenous Parkin as well as overexpressed Parkin and that the S-nitrosylation of endogenous Parkin maintains mitochondrial function and protects cells from cell death due to mitochondrial depolarization. These findings indicate that the activity of Parkin is regulated by both S-nitrosylation and phosphorylation and that defects in either modification can cause ARJP (Fig. 5a).

Although KeyMolnet pathway analysis revealed that differentially expressed proteins in brain tissues from DJ-1^−/−^ mice were associated with ROS signaling (Fig. 1), flow cytometry analysis using MitoSOX showed no significant difference in the production of mitochondrial superoxide between wild-type and DJ-1^−/−^ cells (Supplemental Fig. 4c). In mammalian cells, ROS can cause somatic mutations in mitochondrial DNA, which accumulate throughout life^24^. These mutations then result in impaired function of the respiratory chain, leading to increased ROS production and subsequent accumulation of additional mutations in mitochondrial DNA. However, transformed cells have been reported to exhibit lower frequencies of rare mutations in whole mitochondrial DNA than normal stem cells^25^, suggesting that transformed cells might have an enhanced ROS defense mechanism and do not accumulate mitochondrial DNA mutations. Thus, mitochondrial dysfunction caused by DJ-1 deletion might lead to ROS accumulation in brain tissues from mice but not in SH-SY5Y cells. In addition, mitochondrial ROS have been reported to change activity and expression of proteins involved in glycolysis^26^, suggesting that mitochondrial ROS alter the expression of proteins involved in glycolysis in brain tissues from mice but not SH-SY5Y cells.

DJ-1 has been reported to be S-nitrosylated; however, there is a discrepancy in the S-nitrosylated cysteine(s). Iwatsubo’s group showed that the Cys46 and Cys53 residues, but not the Cys106 residue of DJ-1, are S-nitrosylated^27^, and Lipton’s group showed that the Cys106 residue of DJ-1 is S-nitrosylated, promoting the S-nitrosylation of phosphatase and tensin homolog (PTEN)^28^. We showed that overexpression of wild-type DJ-1, but not C106S mutant DJ-1, in DJ-1^−/−^ cells rescues Parkin S-nitrosylation, suggesting that the transnitrosylation reaction might be a conceivable candidate for the molecular mechanism of Parkin and PTEN S-nitrosylation. However, C106S mutant DJ-1 is S-nitrosylated in wild-type SH-SY5Y cells (Supplemental Fig. S5), and additional investigations are needed to clarify the molecular mechanism of Parkin S-nitrosylation.

The S-nitrosylation of Parkin has a dual role in its activation and inactivation of the protection of mitochondrial function^22,29–31^. Although the S-nitrosylation of the Cys323 residue of Parkin promotes mitochondrial quality control^22^, excess nitric oxide (NO) production inhibits it^31^. In prior studies, drugs, including S-nitrosoglutathione and S-nitrosocysteine, and gene manipulation, including the deletion of S-nitrosoglutathione reductase (GSNOR), have been used to detect S-nitrosylation^29–31^, possibly because of the challenges of S-nitrosylation detection, which can produce excess NO. Importantly, inconsistent with prior studies, the endogenous S-nitrosylation of Parkin was detected in samples from SH-SY5Y cells and mouse brain tissues without enhancement by drugs or gene manipulation in this study (Figs. 3i–l and 4a,b). Although physiological NO activates the E3 ligase activity of Parkin through the S-nitrosylation of Cys323 (Fig. 5a), excess NO/SNOs might pathologically S-nitrosylate one or more cysteine residue(s) other than Cys323, some of which are involved in zinc coordination in Parkin^22^, leading to irreversible conformational disruption and inactivation of Parkin (Fig. 5b). Furthermore, Meng *et al*. reported that the oxidation of Cys253 in Parkin decreases parkin E3 ligase activity^32^. Cys253 is a cysteine residue involved in zinc coordination in the RING1 domain^22^, and thus the oxidation of Cys253 should also cause the irreversible disruption of Parkin conformation. Further investigations are needed to show that pathological oxidative modification of Parkin under pathological conditions contributes to the pathogenesis of sporadic PD.

S-nitrosylation, the reversible covalent addition of a nitrogen monoxide moiety to the thiol side chain of cysteine, like other posttranslational modifications, including phosphorylation and ubiquitination, is an important regulatory mechanism^33^. In addition to finding that the role of S-nitrosylation in the activation of Parkin is complementary to that of phosphorylation, we showed that S-nitrosylation and phosphorylation play complementary roles in the regulation of β-arrestins, which are key proteins for receptor internalization following ligand binding^34^. The stimulus-coupled dephosphorylation of the Ser412 residue of β-arrestin 1 is a prerequisite for clathrin binding and receptor internalization^35^, whereas β-arrestin 2 does not contain an equivalent C-terminal Ser residue but contains the highly conserved cysteine residue Cys410, which is S-nitrosylated; the S-nitrosylation of Cys410 has an effect similar to that of the dephosphorylation of the Ser412 residue of β-arrestin 1^34^, indicating that the S-nitrosylation of β-arrestin 2 and the dephosphorylation of β-arrestin 1 regulate stimulus-induced receptor trafficking in a similar manner.

Based on our findings, we suggest that DJ-1 regulates the activity of Parkin through S-nitrosylation, which plays an important role in the maintenance of mitochondrial function, possibly via ubiquitination of mitochondrial membrane proteins. Moreover, many studies have suggested that excessive or abnormal production of NO plays a crucial role in neuronal cell death associated with neurodegenerative disorders such as PD^36,37^, at least in part, through S-nitrosylation of PD-related proteins, including dynamin-related protein 1 (DRP1)^38,39^, X-chromosome-linked inhibitor of apoptosis protein (XIAP)^40,41^, PTEN^28^, ubiquitin C-terminal hydrolase-L1 (UCHL1)^42^ and PINK1^43^. Our findings support the idea that the activation of Parkin through the physiological S-nitrosylation of Cys323 might provide a novel therapeutic modality. However, as NO could exacerbate PD via Parkin inactivation by pathological S-nitrosylation, the development of tools to upregulate physiological S-nitrosylation but not pathological S-nitrosylation might be required to successfully achieve this goal.

## Materials and Methods

### Approval

All methods were carried out in accordance with relevant guidelines and regulations. All experimental protocols were approved by the institutional committees.

### Animals

DJ-1^−/−^ mice have been described previously (The Jackson Laboratory, Bar Harbor, ME)^44^. The procedures involving animal care and sample preparation were approved by the Animal Experimental Committee of the Juntendo University Graduate School of Medicine and were performed in accordance with the NIH guidelines and regulations and guidelines for the care and use of laboratory animals at the Juntendo University Graduate School of Medicine.

### Proteome analysis and pathway analysis

Proteome analysis and pathway analysis were performed as described previously^45^. Detailed protocols of two-dimensional fluorescence difference gel electrophoresis (2D-DIGE), protein identification, and pathway analysis are described in the Supplemental Materials and Methods.

### Cell culture and transfection

Cells were maintained in Dulbecco’s modified Eagle’s medium supplemented with 10% fetal bovine serum (FBS), 100 U/mL penicillin and 100 µg/mL streptomycin at 37 °C in a humidified 5% CO_2_ atmosphere. SH-SY5Y cells were transfected at 80% confluency using LipofectAMINE LTX (Invitrogen, Carlsbad, CA) according to the manufacturer’s instructions.

### Confocal microscopy

Experiments using confocal microscopy and the calculation of the compaction index were performed as described previously^15,22^. Detailed protocols are described in the Supplemental Materials and Methods.

### Trypan blue viability test

To assess cell viability, cell samples were incubated in trypan blue dye in an acid azo exclusion medium by preparing a 1:1 dilution of cell suspension to 0.4% trypan blue solution. The cells were incubated for 1 min at room temperature and counted under a microscope with a hemocytometer.

### Lactate dehydrogenase (LDH) assay

To assess cell death, the release of LDH into the culture medium was measured using a Cytotoxicity Detection Kit according to the manufacturer’s protocol (Roche Diagnostics, Germany).

### Immunoblot analysis

Immunoblotting was performed as described previously^22^. Detailed protocols are described in the Supplemental Materials and Methods. The intensities of the band on the immunoblots were quantified by densitometry using NIH ImageJ software. Uncropped images of the immunoblots are shown in Supplemental Fig. S8.

### Detection of S-nitrosylated proteins by SNO-RAC

S-nitrosylated proteins were detected by using the SNO-RAC method as previously described^23^ with some modifications^22^. Detailed protocols are described in the Supplemental Materials and Methods.

### ATP evaluation

Quantitative determination of ATP was performed using an ATP Bioluminescence Assay Kit HS II (Roche Diagnostics, Germany) according to a protocol defined in a previous manuscript. In brief, cells diluted to a concentration of 10^5^ cells/mL were added to equal volumes of cell lysis reagent and incubated first for 5 min at room temperature and then for another 2 min at 100 °C. The samples were centrifuged for 1 min, and the supernatants were transferred to fresh tubes. Luciferase reagent was added to the samples, and the samples were measured using a luminometer (Promega, WI).

### Measurement of the cellular OCR and ECAR

For analyses of mitochondrial respiration and glycolytic function, the cellular OCR and ECAR were measured using an XFp extracellular flux analyzer and an XFp extracellular flux cartridge (Seahorse Bioscience, North Billerica, MA) in combination with an XFp Cell Mito Stress Test Kit and XFp Glycolysis Stress Test Kit, respectively^21^. Cells were plated in an XFp miniplate (Seahorse Bioscience) at a concentration of 3.0–4.0 × 10^4^ cells/well (three wells/sample) and incubated at 37 °C for 24 h in a 5% CO_2_ atmosphere. The medium was changed to assay medium, and the cells were incubated at 37 °C for 1 h in a CO_2_-free atmosphere. The composition of the assay medium was as follows: XF base medium containing 10 mM glucose, 1 mM sodium pyruvate, and 2 mM Gluta Max (pH 7.4, 37 °C) for the measurement of the OCR (Mito Stress Test) and XF base medium containing 2 mM L-glutamine (pH 7.4, 37 °C) for the measurement of the ECAR (Glycolysis Stress Test). The OCR was measured under basal conditions and in response to 2.5 μM oligomycin, 1 μM carbonyl cyanide-p-trifluoromethoxyphenylhydrazone (FCCP), and 1 μM rotenone/1 μM antimycin A. ECAR was measured under basal conditions and in response to 10 mM glucose, 2.5 μM oligomycin, and 50 mM 2-deoxy-D-glucose (2-DG). After the assay, the supernatant was removed, and protein extraction buffer (1 × Laemmli Sample Buffer (BIO-RAD) containing 50 mM DTT, 25 μL/well) was added to the cells. The protein concentration was determined using Pierce660 nm Protein Assay Reagent in combination with Ionic Detergent Compatibility Reagent (IDCR). The OCR and ECAR values were normalized to the amount of total protein. Three experiments were performed, and the data were averaged.

## Supporting Information

Supporting Information.
