# Supplementary material for: DJ-1 is indispensable for the S-nitrosylation of Parkin, which maintains function of mitochondria
Source: Sci Rep. 2020 Mar 9;10:4377. doi: 10.1038/s41598-020-61287-6 (PMC7062835; doi:10.1038/s41598-020-61287-6)
Supplement: Supplementary file 1 — Supporting Information. [file 41598_2020_61287_MOESM1_ESM.pdf]

DJ-1 is indispensable for the S-nitrosylation of Parkin, which maintains function of mitochondria.

Kentaro Ozawa[1, 2, 3, 4, 5*], Hiroki Tsumoto[1, 2], Yuri Miura[1, 2], Junji Yamaguchi[4], Sanae M. M. Iguchi-Ariga[6], Tetsushi Sakuma[7], Takashi Yamamoto[7], Yasuo Uchiyama[4]

1. Research Team for Mechanism of Aging, Tokyo Metropolitan Institute of Gerontology, 35-2 Sakae-cho, Itabashi-ku, Tokyo 173-0015, Japan.
2. These authors contributed equally to this work.
3. Department of Pharmacology, Nara Medical University School of Medicine, Kashihara City, Nara, 634-8521, Japan.
4. Department of Cellular and Molecular Neuropathology, Juntendo University Graduate School of Medicine, Bunkyo-Ku 2-1-1, Tokyo, 113-8421, Japan.
5. Asakayama General Hospital, Sakai-ku, Sakai City, Osaka, 590-0018, Japan
6. Faculty of Pharmaceutical Sciences, Hokkaido University, Kita 12, Nishi 6, Kita-ku, Sapporo, 060-0812, Japan.
7. Division of Integrated Sciences for Life, Graduate School of Integrated Sciences for Life, Hiroshima University, Hiroshima, 739-8526, Japan.

Corresponding Author: Kentaro Ozawa, M.D., Ph.D.

Research Team for Mechanism of Aging, Tokyo Metropolitan Institute of Gerontology, 35-2 Sakae-cho, Itabashi-ku, Tokyo 173-0015, Japan

TEL: +81-3-3964-3241

FAX: +81-3-3579-4776

E-mail: ozawa.sno.man@gmail.com

Supplemental Information

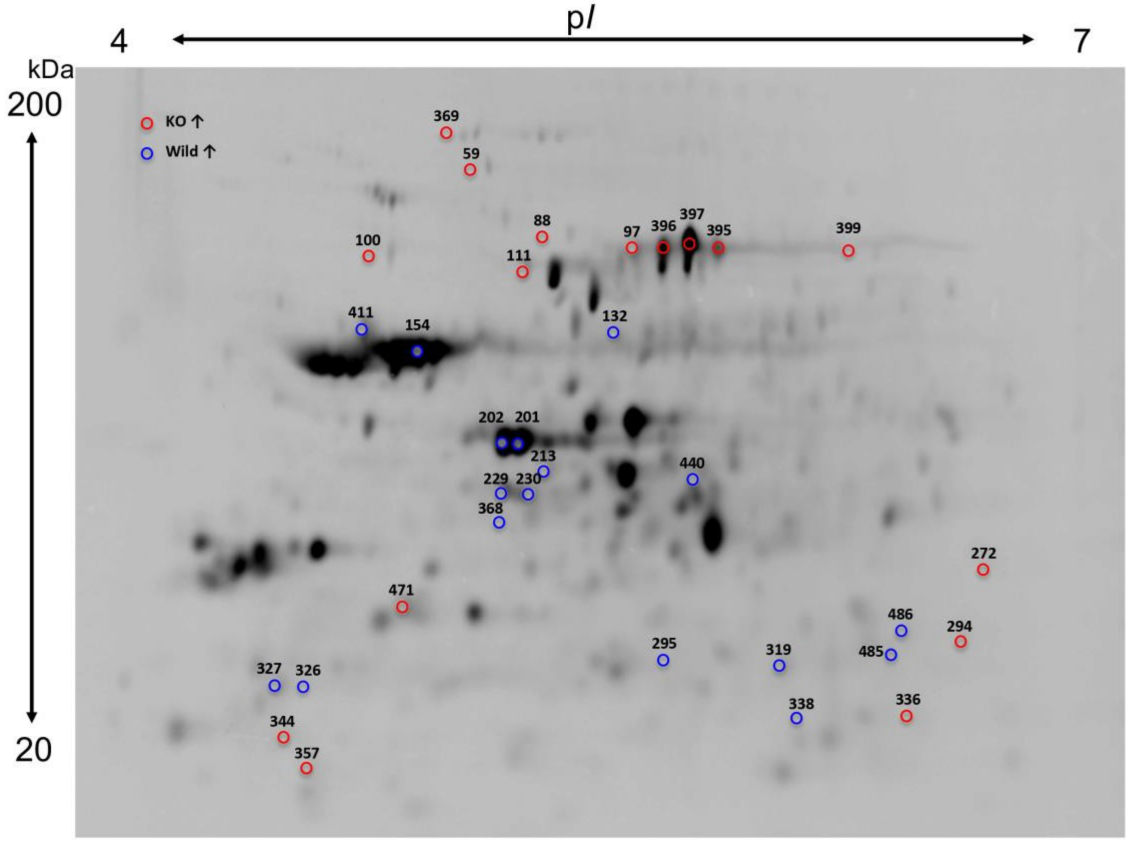

Supplemental Figure S1. Location of selected spots on the 2D images.
Thirty-three protein spots whose intensities changed in brain tissues of DJ-1-/- mice are labeled on images of 2D gels. Number represents ID of each spot as assigned by Progenesis SameSpots (ver. 4.5). Data of each spot are summarized in Table S1.

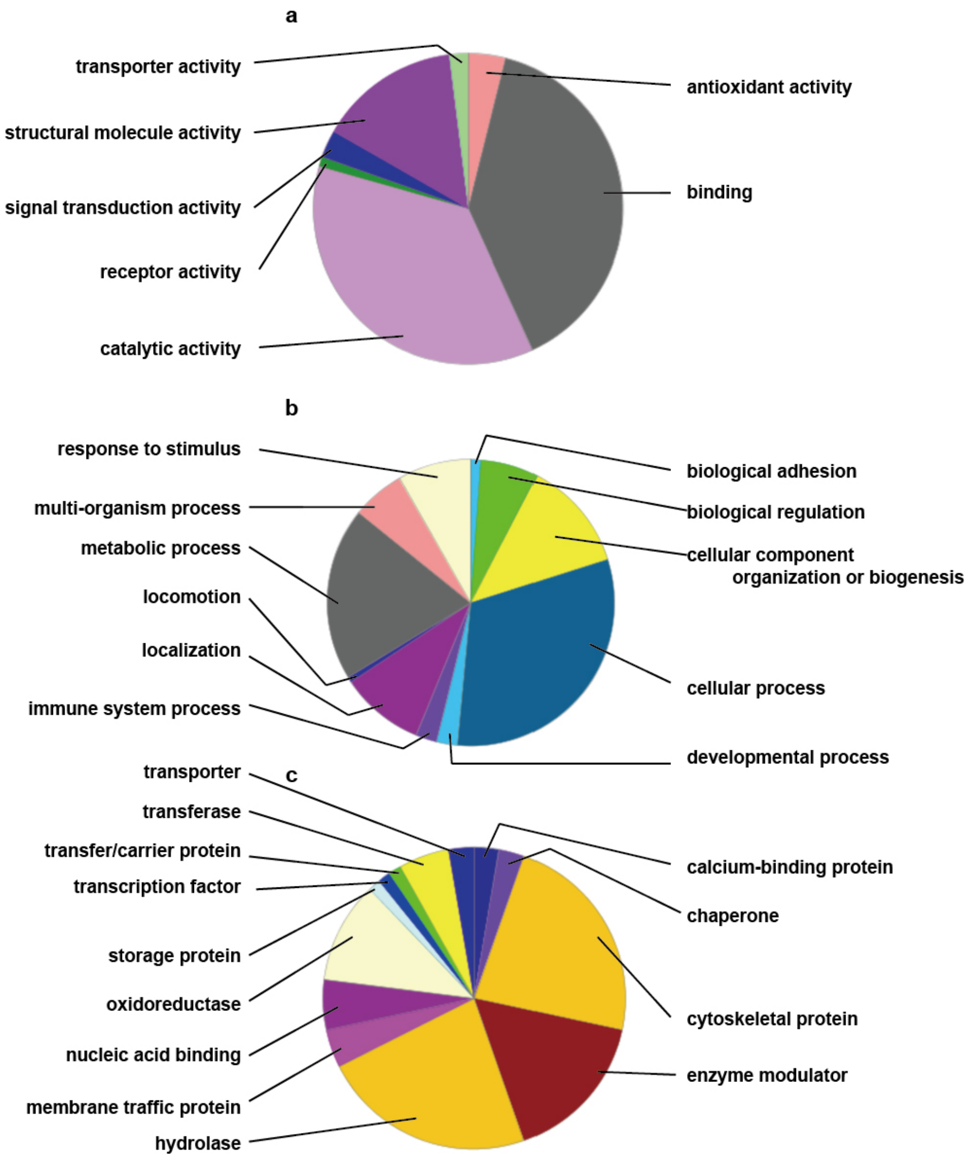

Supplemental Figure S2. Categorization of the differentially expressed proteins in the brain tissues of DJ-1-/- mice.

The differentially expressed proteins that are identified by MIS can be classified into 7 molecular functional categories (a), 11 biological processes (b), and 13 protein classes (c) by the PANTHER Classification System.

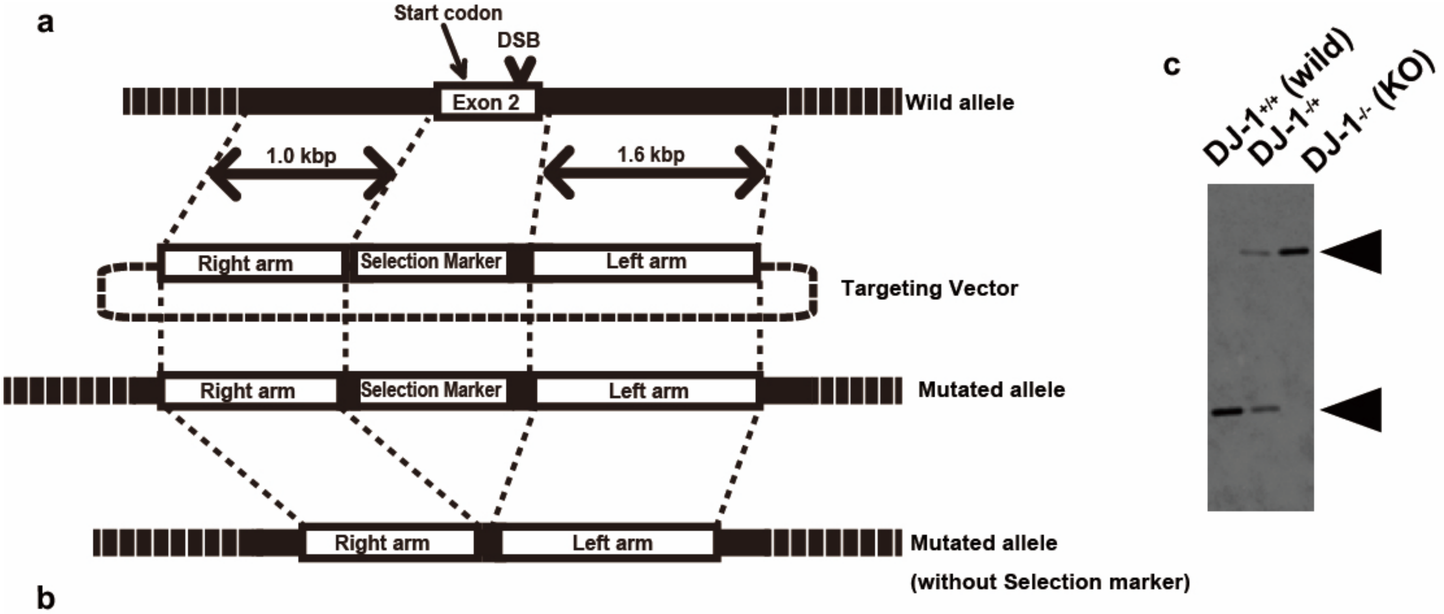

b

gggtatctcagggttgcaatgaaagtttttgaaatcttttttttttttttttttaag
gcttgtaaacatataacataaaaATGgcttccaaaagagctctggtcatcctggctaa
aggagcagaggaaatggagacggtcatccctgtagatgtcatgaggcgagctggggtaa
gtcccacatcgattttagccattcc

Supplemental Figure S3. Generation of SH-SY5Y cells deleted with DJ-1 gene.

(a) Schematic representation of wild-type, mutant alleles of DJ-1 gene. DSB represents double strand break. (b) The partial sequence of DJ-1 gene. The red characters and the highlighted in green represent sequences of exon with the first codon (squared) and TALEN respectively. (c) Southern blotting of genomic DNA of wild-type SH-SY5Y cells, heterozygous (DJ-1$^{-/+}$ cells) and homozygous (DJ-1$^{-/-}$ cells) with selection marker.

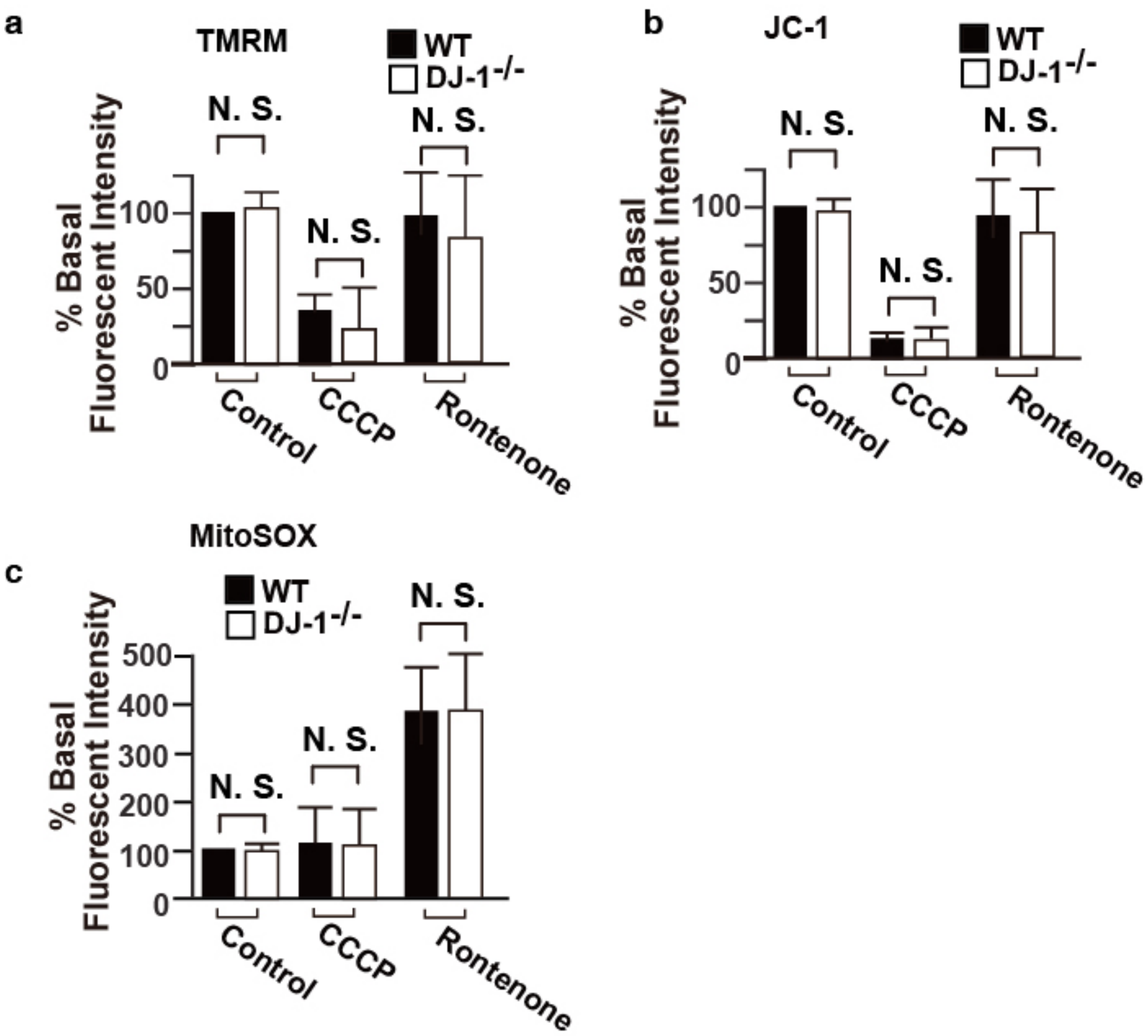

Supplemental Figure S4. Evaluation of mitochondrial membrane potential and superoxide of wild-type and DJ-1$^{-/-}$ cells by flow cytometry analysis.

Wild-type and DJ-1$^{-/-}$ cells under normal condition or with CCCP/ rotenone were incubated with TMRM, JC-1 or MitoSOX, followed by flow cytometry analysis. Data shown are mean ± SE (n=3); N.S. means no significant difference.

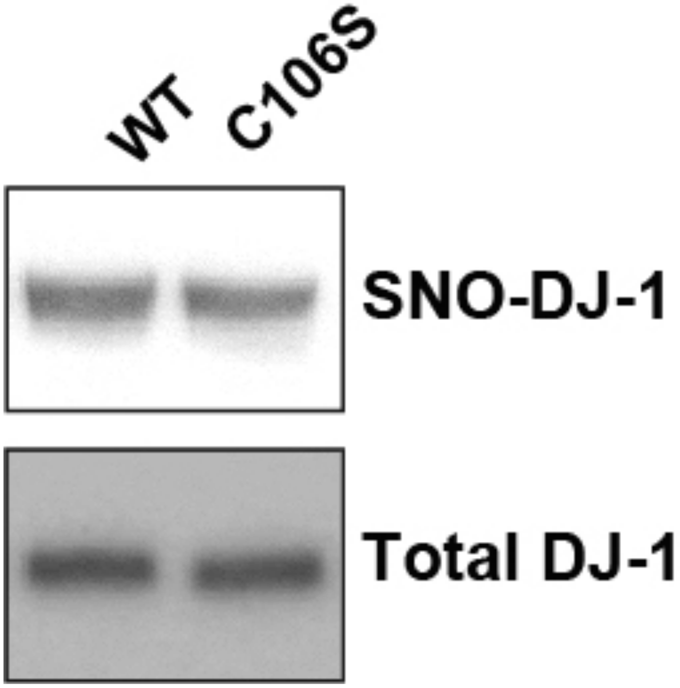

Supplemental Figure S5. Evaluation of S-nitrosylation of wild-type and C106S DJ-1 in SH-SY5Y cells. Wild-type SH-SY5Y cells were transfected with the FLAG-tagged wild-type or C106S DJ-1 plasmid and were then incubated for 48 h. Lysates were used for immunoblot analysis with anti-FLAG antibody (total DJ-1) and analysis by SNO-RAC, in which ascorbate-dependent purification demonstrates the presence of S-nitrosylated cysteine residues, with subsequent immunoblot analysis with the anti-FLAG antibody (SNO-DJ-1), as indicated.

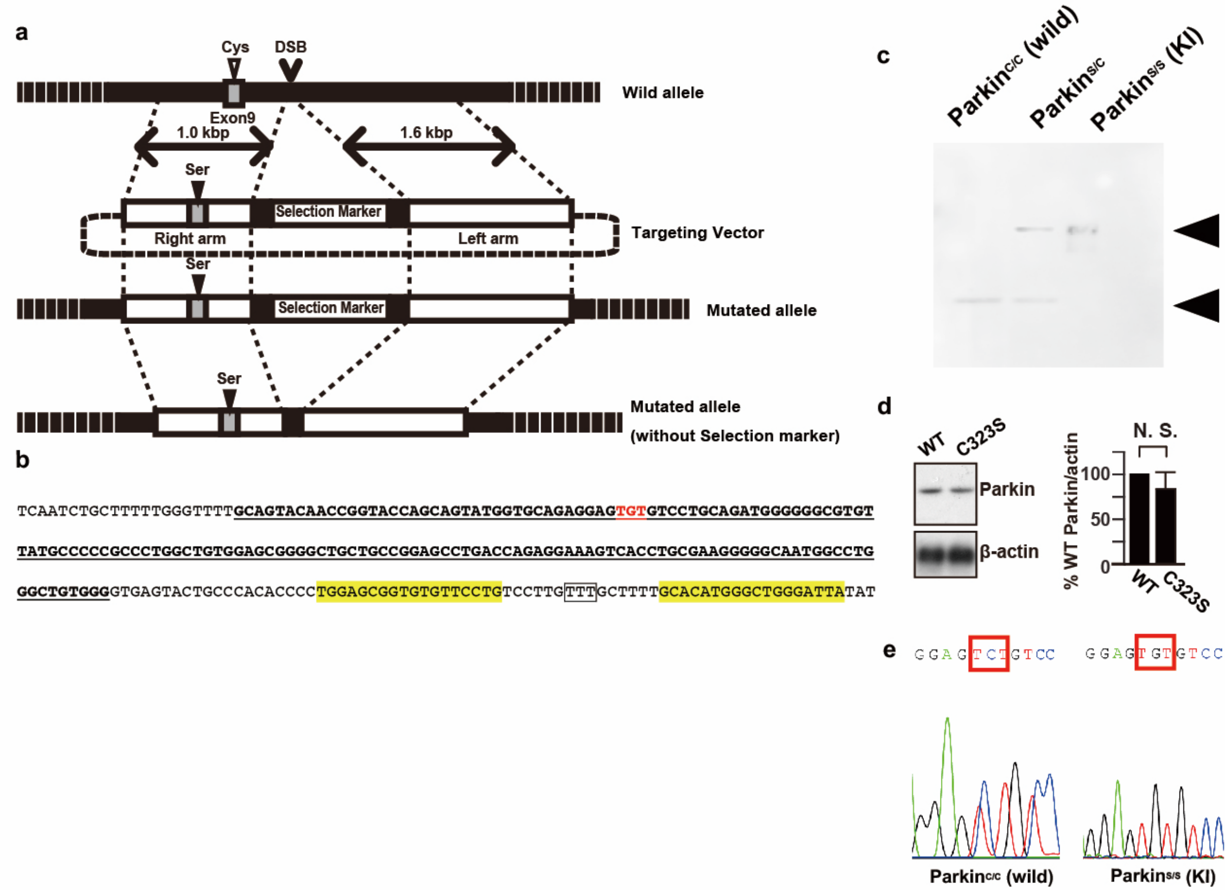

Supplemental Figure S6. Generation of SH-SY5Y cells with the mutation of Cys323 in parkin converted to serine. (a) Schematic representation of wild-type, mutant alleles of parkin gene. DSB represents double strand break. Cys represents Cys 323 in parkin gene. (b) The partial sequence of parkin gene. The red characters and the highlighted in green represent sequences coding Cys323 and TALEN respectively. Bold characters with underline represent sequences of exon. The box represents sequences of DSB. (c) Southern blotting of genomic DNA of wild-type SH-SY5Y cells, heterozygous (parkin$^{S/C}$) and homozygous (parkin$^{S/S}$) mutated cells with selection marker. Homozygous (parkin$^{S/S}$) mutated cells are equal to parkin^{C323S} cells. (d) Immunoblot analysis of cell lysates from wild-type SH-SY5Y and parkin^{C323S} cells with anti-parkin (upper panel) and anti-beta-actin (lower panel) antibodies. The quantity of parkin, as measured by scanning densitometry, is expressed as a percentage of wild-type cells, normalized with respect to β-actin (lower panels). Data shown are mean ± SE (n = 3); N.S. means no significant difference. (e) The DNA base sequence of cDNA purified from wild-type and parkin^{C323S} cells by the Sanger's method.

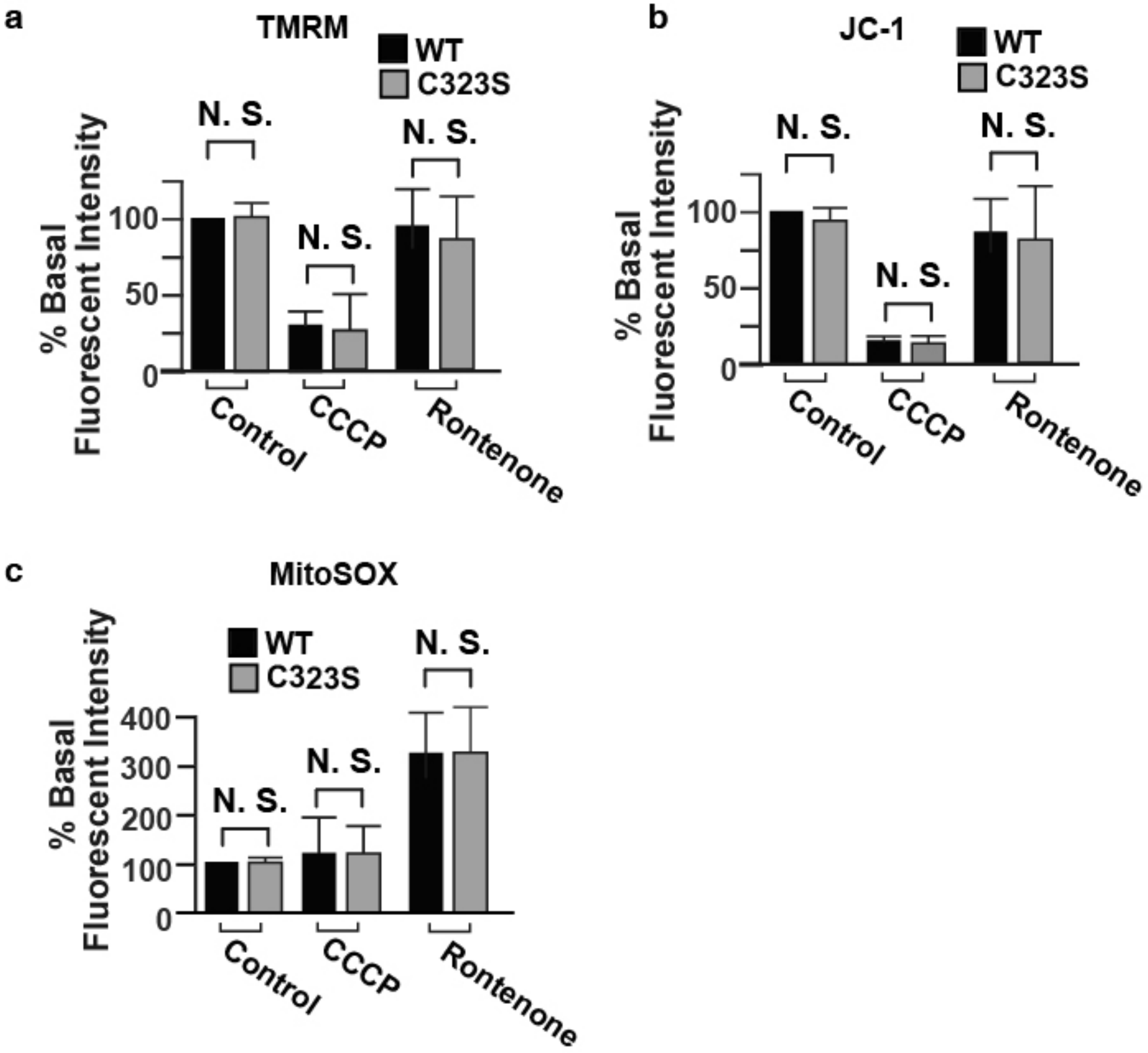

Supplemental Figure S7. Evaluation of mitochondrial membrane potential and superoxide of wild-type and parkin^{C323S} cells by flow cytometry analysis.

Wild-type and parkin^{C323S} cells under normal condition or with CCCP/ rotenone were incubated with TMRM, JC-1 or MitoSOX, followed by flow cytometry analysis. Data shown are mean ± SE (n=3); N.S. means no significant difference.

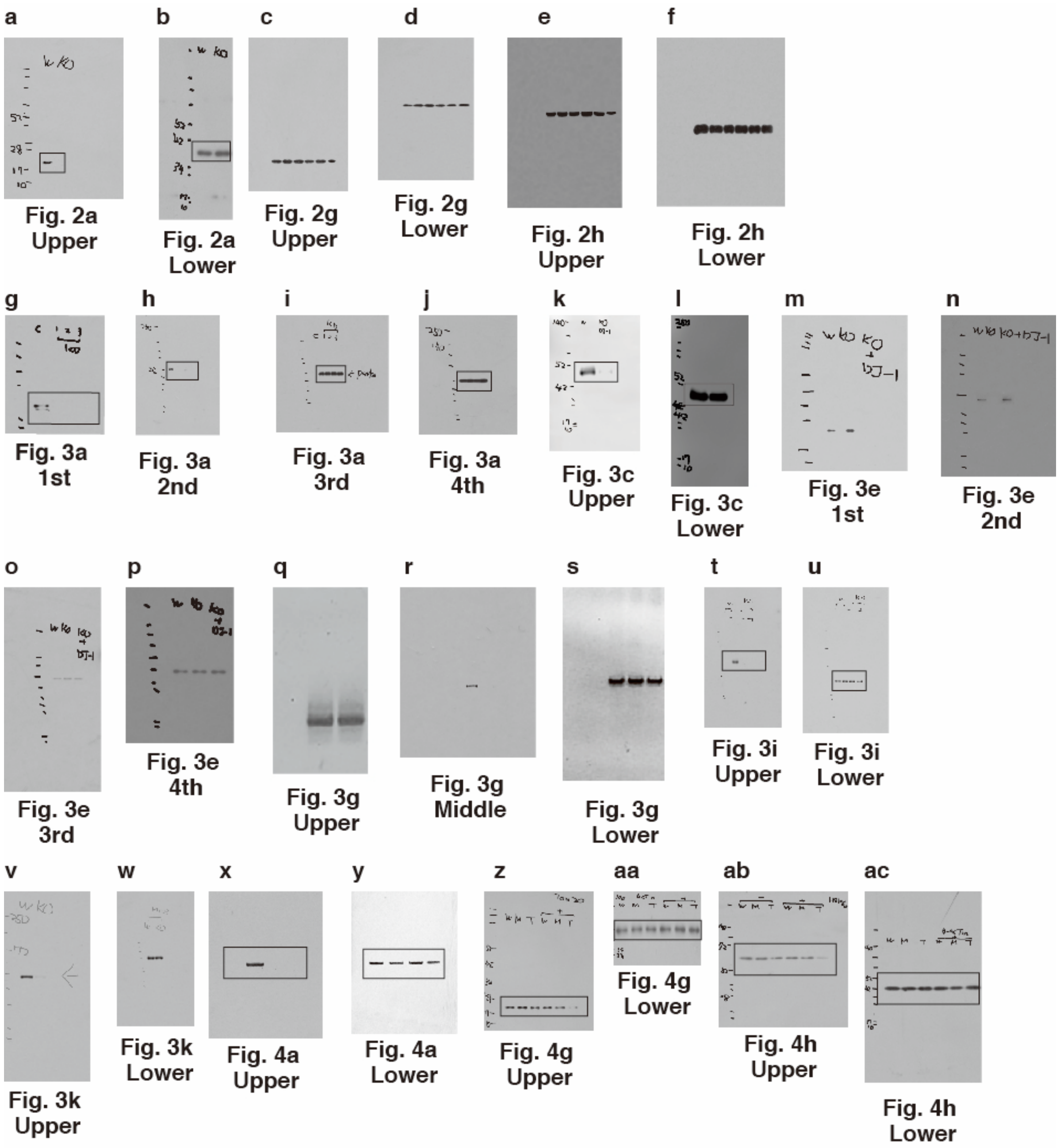

Supplemental Figure S8. Uncropped pictures of immunoblotting.

Uncropped pictures were shown as indicated.

Supplemental Table S1 Spot analysis and protein identification of differentially expressed proteins in DJ-1 knockout mice

Spot analysis (Progenesis SameSpots)					Protein identification (Protein Pilot™)					
Spot No.[a]	Anova (p)	Fold	Highest Mean	Area	Unused[b]	Protein Accession[c]	Gene Name	Protein Description	Peptides (95%)[d]	Coverage (%)[e]
59	0.0009	1.37	KO	167	40.81	Q01853	Vcp	Transitional endoplasmic reticulum ATPase	19	27.79
88	0.0343	1.33	KO	215	12.76	P14824	Anxa6	Annexin A6	6	9.36
97	0.0328	1.40	KO	632	56.01	P07724	Alb	Serum albumin	27	47.37
					14.14	P38647	Hspa9	Stress-70 protein, mitochondrial	7	14.29
					13.3	P63017	Hspa8	Heat shock cognate 71 kDa protein	7	13.62
100	0.0013	1.18	KO	308	42.26	P20029	Hspa5	78 kDa glucose-regulated protein	21	42.14
111	0.0013	1.24	KO	623	80.51	P63017	Hspa8	Heat shock cognate 71 kDa protein	55	61.61
					4.12	P50516	Atp6v1a	V-type proton ATPase catalytic subunit A	3	7.13
132	0.0142	1.15	WT	229	25.72	P63328	Ppp3ca	Serine/threonine-protein phosphatase 2B catalytic subunit alpha isoform	12	25.91
					14.17	P63038	Hspd1	60 kDa heat shock protein, mitochondrial	7	18.15
					10.53	P68373	Tuba1c	Tubulin alpha-1C chain	5	14.92
					6.9	P61979	Hnrnpk	Heterogeneous nuclear ribonucleoprotein K	3	8.21
					4.05	P07724	Alb	Serum albumin	2	4.44
					4	P42932	Cct8	T-complex protein 1 subunit theta	2	3.83
					3.16	Q9CYT6	Cap2	Adenylyl cyclase-associated protein 2	2	4.20
					2	P68369	Tuba1a	Tubulin alpha-1A chain	5	14.86
154	0.0096	1.09	WT	411	75.77	P05213	Tuba1b	Tubulin alpha-1B chain	68	76.5
					21.39	P56480	Atp5f1b	ATP synthase subunit beta, mitochondrial	11	32.89
					14.9	Q9ERD7	Tubb3	Tubulin beta-3 chain	7	19.33
					6	P68368	Tuba4a	Tubulin alpha-4A chain	60	65.85
					3.38	P68369	Tuba1a	Tubulin alpha-1A chain	70	76.50
					2.86	P14211	Calr	Calreticulin	2	6.01
					2.29	Q7TMM9	Tubb2a	Tubulin beta-2A chain	7	19.33
201	0.0480	1.12	WT	638	63.37	P63260	Actg1	Actin, cytoplasmic 2	59	75.73
					16.87	Q62420	Sh3gl2	Endophilin-A1	9	29.26
					4	P07724	Alb	Serum albumin	2	4.77
					2	P60710	Actb	Actin, cytoplasmic 1	55	75.73
					2	P68134	Acta1	Actin, alpha skeletal muscle	25	31.83
202	0.0054	1.16	WT	776	65.5	P63260	Actg1	Actin, cytoplasmic 2	60	73.6
					10	Q62420	Sh3gl2	Endophilin-A1	5	20.17
					2	P60710	Actb	Actin, cytoplasmic 1	65	73.60
213	0.0251	1.14	WT	308	20.45	Q9D819	Ppa1	Inorganic pyrophosphatase	10	44.98
					20.31	P62874	Gnb1	Guanine nucleotide-binding protein G(I)/G(S)/G(T) subunit beta-1	10	34.12
					17.47	P63260	Actg1	Actin, cytoplasmic 2	9	30.40
					12.09	P68373	Tuba1c	Tubulin alpha-1C chain	7	19.15
					5.85	P62880	Gnb2	Guanine nucleotide-binding protein G(I)/G(S)/G(T) subunit beta-2	7	25
229	0.0379	1.16	WT	611	36	P18872	Gnao1	Guanine nucleotide-binding protein G(o) subunit alpha	22	57.34
					15.47	P63260	Actg1	Actin, cytoplasmic 2	8	29.60
					10	Q9D6R2	Idh3a	Isocitrate dehydrogenase [NAD] subunit alpha, mitochondrial	5	13.66
					4.99	Q64674	Srm	Spermidine synthase	3	11.59
					4.77	Q9QYG0	Ndrg2	Protein NDRG2	3	8.09
					4	Q9DB05	Napa	Alpha-soluble NSF attachment protein	2	7.46
					4	P28663	Napb	Beta-soluble NSF attachment protein	2	7.38
230	0.0298	1.13	WT	402	34	P28663	Napb	Beta-soluble NSF attachment protein	21	62.08
					18.7	P18872	Gnao1	Guanine nucleotide-binding protein G(o) subunit alpha	9	30.51
					18.01	P60710	Actb	Actin, cytoplasmic 1	9	32.53
					16.98	Q9D6F9	Tubb4a	Tubulin beta-4 chain	8	24.10
					14	Q9D6R2	Idh3a	Isocitrate dehydrogenase [NAD] subunit alpha, mitochondrial	7	20.49
					12.24	P68373	Tuba1c	Tubulin alpha-1C chain	7	19.15
					11.4	Q9DB05	Napa	Alpha-soluble NSF attachment protein	10	37.29
					6	Q7TMM9	Tubb2a	Tubulin beta-2A chain	6	17.30
					4.78	Q04447	Ckb	Creatine kinase B-type	4	11.02
					4.03	Q8VDQ1	Ptgr2	Prostaglandin reductase 2	2	7.98
					3.29	Q9D051	Pdhb	Pyruvate dehydrogenase E1 component subunit beta, mitochondrial	2	7.52
					2.46	Q64674	Srm	Spermidine synthase	2	8.28
272	0.0022	1.27	KO	788	16.31	Q9QUM9	Psma6	Proteasome subunit alpha type-6	8	42.68
					12	Q9DBJ1	Pgam1	Phosphoglycerate mutase 1	6	36.22
					4	Q9DCM0	Ethe1	Protein ETHE1, mitochondrial	2	9.05
294	0.0223	1.26	KO	777	20	O08709	Prdx6	Peroxiredoxin-6	10	53.57
					2.53	P97823	Lypla1	Acyl-protein thioesterase	2	10.87
295	0.0440	1.13	WT	653	7.07	P09528	Fth1	Ferritin heavy chain	3	18.13
					4	Q9R0Y5	Ak1	Adenylate kinase isoenzyme 1	2	13.92
					4	P56480	Atp5f1b	ATP synthase subunit beta, mitochondrial	2	4.92
319	0.0129	1.15	WT	720	12	P60766	Cdc42	Cell division control protein 42 homolog	6	35.08
326	0.0262	1.20	WT	299	6	Q80ZJ1	Rap2a	Ras-related protein Rap-2a	3	20.77
					4.97	P84075	Hpca	Neuron-specific calcium-binding protein hippocalcin	3	17.10
					2	P61226	Rap2b	Ras-related protein Rap-2b	2	14.21
327	0.0464	1.24	WT	770	20	P84075	Hpca	Neuron-specific calcium-binding protein hippocalcin	11	59.07
					6	O55042	Snca	Alpha-synuclein	3	36.43
					4	P61226	Rap2b	Ras-related protein Rap-2b	2	12.02
					3.31	P63028	Tpt1	Translationally-controlled tumor protein	2	13.37
336	0.2480	1.54	KO	968	10	P08228	Sod1	Superoxide dismutase [Cu-Zn]	5	37.01
338	0.0350	1.16	WT	346	8.73	P56380	Nudt2	Bis(5'-nucleosyl)-tetraphosphatase [asymmetrical]	4	33.33
					3.19	P61089	Ube2n	Ubiquitin-conjugating enzyme E2 N	2	13.82
					2.93	P97478	Coq7	Ubiquinone biosynthesis protein COQ7 homolog	2	11.98
					1.82	Q922U2	Krt5	Keratin, type II cytoskeletal 5	3	3.45
344	0.4417	1.46	KO	480	18	Q9CWF2	Tubb2b	Tubulin beta-2B chain	10	25.39
					4	P56395	Cyb5a	Cytochrome b5	2	18.66
					3.06	P68372	Tubb4b	Tubulin beta-2C chain	5	15.73
					2	Q7TMM9	Tubb2a	Tubulin beta-2A chain	10	25.39
					2	P99024	Tubb5	Tubulin beta-5 chain	7	15.77
357	0.1144	1.47	KO	582	12	Q9CWF2	Tubb2b	Tubulin beta-2B chain	6	21.80
					7.8	Q62048	Pea15	Astrocytic phosphoprotein PEA-15	4	35.38
					6	Q9D6F9	Tubb4a	Tubulin beta-4 chain	4	12.61
					4.54	P32848	Pvalb	Parvalbumin alpha	3	34.55
					2	Q7TMM9	Tubb2a	Tubulin beta-2A chain	6	21.80
					2	P99024	Tubb5	Tubulin beta-5 chain	5	15.77
					2	Q9ERD7	Tubb3	Tubulin beta-3 chain	4	13.78
368	0.0003	1.15	WT	449	21.7	P62715	Ppp2cb	Serine/threonine-protein phosphatase 2A catalytic subunit beta isoform	12	46.28
					17.54	O08553	Dpysl2	Dihydropyrimidinase-related protein 2	9	23.60
					8.56	Q61166	Mapre1	Microtubule-associated protein RP/EB family member 1	4	20.15
					8	P10605	Ctsb	Cathepsin B	4	16.81
					8	Q99M71	Epdr1	Mammalian ependymin-related protein 1	4	21.88
					5.93	P56480	Atp5f1b	ATP synthase subunit beta, mitochondrial	3	7.56
					2.18	Q6PER3	Mapre3	Microtubule-associated protein RP/EB family member 3	3	12.46
					2	P63330	Ppp2ca	Serine/threonine-protein phosphatase 2A catalytic subunit alpha isoform	12	46.28
369	0.0343	1.15	KO	317	38.84	Q61316	Hspa4	Heat shock 70 kDa protein 4	20	31.75
					13.16	Q6IFX2	Krt42	Keratin, type I cytoskeletal 42	7	15.49
					10.01	Q3TTY5	Krt2	Keratin, type II cytoskeletal 2 epidermal	13	16.83
					5.58	Q922U2	Krt5	Keratin, type II cytoskeletal 5	11	14.66
					4.01	Q02257	Jup	Junction plakoglobin	2	4.43
395	0.00202	1.53	KO	929	57.34	P07724	Alb	Serum albumin	29	51.81
396	0.02072	1.69	KO	786	73.54	P07724	Alb	Serum albumin	40	58.39
					4	Q61696	Hspa1a	Heat shock 70 kDa protein 1A	2	4.37
397	0.00488	1.78	KO	698	75.88	P07724	Alb	Serum albumin	44	64.47
399	0.0070	1.35	KO	432	48.91	P07724	Alb	Serum albumin	26	43.91
					6.26	Q64521	Gpd2	Glycerol-3-phosphate dehydrogenase, mitochondrial	4	6.60
					4.55	P63017	Hspa8	Heat shock cognate 71 kDa protein	2	5.26
					4	Q91X72	Hpx	Hemopexin	2	4.78
411	0.0086	1.12	WT	647	65.71	P50396	Gdi1	Rab GDP dissociation inhibitor alpha	44	69.80
					18.54	Q76MZ3	Ppp2r1a	Serine/threonine-protein phosphatase 2A 65 kDa regulatory subunit A alpha isoform	10	15.79
					15.44	P68373	Tuba1c	Tubulin alpha-1C chain	7	22.27
					12.25	Q9ERD7	Tubb3	Tubulin beta-3 chain	6	16.22
440	0.0313	1.16	WT	248	22.11	Q9D6R2	Idh3a	Isocitrate dehydrogenase [NAD] subunit alpha, mitochondrial	11	33.61
					10.22	P29387	Gnb4	Guanine nucleotide-binding protein subunit beta-4	5	22.35
					8.51	Q7TMM9	Tubb2a	Tubulin beta-2A chain	4	12.13
					8	P68373	Tuba1c	Tubulin alpha-1C chain	4	13.36
					7.22	P62874	Gnb1	Guanine nucleotide-binding protein G(I)/G(S)/G(T) subunit beta-1	5	15.29
					5.96	P08752	Gnai2	Guanine nucleotide-binding protein G(i) subunit alpha-2	3	9.58
					4.01	P50396	Gdi1	Rab GDP dissociation inhibitor alpha	2	7.61
					4	Q9CQT1	Mri1	Methylthioribose-1-phosphate isomerase	2	5.69
471	0.0259	1.07	KO	644	26.41	P70296	Pebp1	Phosphatidylethanolamine-binding protein 1	16	93.05
					8.01	Q8K3J1	Ndufs8	NADH dehydrogenase [ubiquinone] iron-sulfur protein 8, mitochondrial	4	23.11
					6	Q61171	Prdx2	Peroxiredoxin-2	3	18.69
					6	P63054	Pcp4	Purkinje cell protein 4	3	43.55
485	0.00016	1.69	WT	195	14	Q99LX0	Park7	Protein DJ-1	8	41.27
					5.89	P11352	Gpx1	Glutathione peroxidase 1	3	16.42
486	0.121	1.15	WT	320	4.43	Q9R1P1	Psmb3	Proteasome subunit beta type-3	2	12.68

[a] Spot number by Progenesis SameSpots automatically. Position of each spot on 2D-gel is shown in Supplemental Fig. S1.

[b] Protein score (Unused) by ProteinPilot™.

[c] Accession number of UniProt.

[d] Number of peptides with an ion score above 95% confidence.

[e] % coverage by peptides with an ion score above 95% confidence of each protein assigned.

Cut-off values of fold change and p-value, and selection criteria of protein identification were described in Materials and Methods section.

Supplemental Table S3 High relevant networks to DJ-1-deletion and accosiated molecules based on "Interaction search" of KeyMolnet

Network	Symbol[a]	Molecule Name[b]	Name[a]
Glycolytic pathway (Score[c] 41.9)	ADP	ADP	adenosine 5'-diphosphate
	ATP	ATP	adenosine 5'-triphosphate
	GPDH-M	Gpd2	Glycerol-3-phosphate dehydrogenase, mitochondrial
	L-protein	Dld	Dihydrolipoamide dehydrogenase
	p53	Tp53	Cellular tumor antigen p53
	PDH	PDH	Pyruvate dehydrogenase complex
	PyrDHE1a	Pdha	Pyruvate dehydrogenase E1 component alpha subunit
	PyrDHE1b	Pdhb	Pyruvate dehydrogenase E1 component beta subunit
	PyrDHE2	Dlat	Dihydrolipoamide acetyltransferase component of pyruvate dehydrogenase complex
	PyrDHE3BP	Pdhx	Pyruvate dehydrogenase protein X component
Creatine pathway (Score 38.0)	ADP	ADP	adenosine 5'-diphosphate
	ATP	ATP	adenosine 5'-triphosphate
	BCK	Ckb	brain creatine kinase
	CK	Ckb	Creatine kinase
	creatine	creatine	Creatine
	ONOO	ONOO	peroxynitrite
	p53	Tp53	Cellular tumor antigen p53
	PCr	phosphorylcreatine	Phosphorylcreatine
TCA cycle (Score 34.3)	ADP	ADP	adenosine 5'-diphosphate
	ATP	ATP	adenosine 5'-triphosphate
	L-protein	Dld	Dihydrolipoamide dehydrogenase
	PDH	Dlat	Pyruvate dehydrogenase complex
	PyrDHE1a	Pdha	Pyruvate dehydrogenase E1 component alpha subunit
	PyrDHE1b	Pdhb	Pyruvate dehydrogenase E1 component beta subunit
	PyrDHE2	Dlat	Dihydrolipoamide acetyltransferase component of pyruvate dehydrogenase complex
	PyrDHE3BP	Pdhx	Pyruvate dehydrogenase protein X component
ROS signaling pathway (Score 40.6)	c-Abl	Abl1	Proto-oncogene tyrosine-protein kinase ABL1
	Ca2+	Ca2+	calcium ion
	Cdc42	Cdc42	cell division cycle 42
	GPX1	Gpx1	Glutathione peroxidase 1
	H2O2	hydrogen peroxide	hydrogen peroxide
	HSF-1	Hsf1	heat shock transcription factor-1
	NADHDHq	Ndufs8	NADH dehydrogenase [ubiquinone]
	Nrf2	Nfe2l2	Nuclear factor erythroid 2 related factor 2
	PRDX2	Prdx2	Peroxiredoxin 2
	ROS	ROS	reactive oxygen species
Autophagy-related protein signaling pathway (Score 35.3)	c-Src	Src	Proto-oncogene tyrosine-protein kinase Src
	C/EBPb	Cebpb	CCAAT/enhancer binding protein beta
	ErbB1	Egfr	Receptor protein-tyrosine kinase ErbB-1
	ERK	Mapk	extracellular signal regulated kinase
	Nrf2	Nfe2l2	Nuclear factor erythroid 2 related factor 2
	p53	Tp53	Cellular tumor antigen p53
	parkin	Prkn	parkin
	PKC	Prkca	protein kinase C
	SIRT1	Sirt1	NAD-dependent deacetylase sirtuin 1
	STAT3	Stat3	signal transducer and activator of transcription 3
	TRAF6	Traf6	TNF receptor associated factor 6
mitochondrial dynamics (Score 31.6)	a-tub	Tuba1	alpha tubulin
	ADP	ADP	adenosine 5'-diphosphate
	ATP	ATP	adenosine 5'-triphosphate
	CDC48	Vcp	valosin-containing protein
	dynein	Dnah1	dynein
	NADHDHq	Ndufs8	NADH dehydrogenase [ubiquinone]
	parkin	Prkn	parkin
	ROS	ROS	reactive oxygen species
Parkinson diseases (Score 20.5)	a-syn	Snca	alpha-synuclein
	creatine	creatine	Creatine
	DJ-1	Park7	Oncogene DJ1
	NADHDHq	Ndufs8	NADH dehydrogenase [ubiquinone]
	parkin	Prkn	parkin

a Symbol and Name are according to KeyMolnet.
b Molecule name corresponds to Fig. 1.
c Score is calculated by "Interaction search" algorism by KeyMolnet.

Supplemental Materials and Methods

Materials

An Immobiline DryStrip gel (pH 4-7, 18 cm long) and Pharmalyte (pH 3-10) were purchased from GE Healthcare (Uppsala, Sweden). Tris, tricine, sodium dodecyl sulfate (SDS), thiourea, ammonium bicarbonate, Triton X-100, and alpha-cyano-4-hydroxycinnamic acid (CHCA, MALDI-MS grade) were acquired from Sigma-Aldrich (St. Louis, MO, USA). Dithiothreitol (DTT) and ammonium persulfate were purchased from Nacalai Tesque (Kyoto, Japan). Acetonitrile (MeCN) containing 0.1% trifluoroacetic acid (TFA) (LC-MS grade) and water containing 0.1% TFA (LC-MS grade) were acquired from Thermo Fisher Scientific Inc. (Rockford, IL, USA). 3-[(3-Cholamidopropyl) dimethylammonio]-1-propanesulfonate (CHAPS), 4-(2-hydroxyethyl)-1-piperazineethanesulfonic acid (HEPES), *N*-ethyl-*N*′-[5-(*N*″-succinimidyloxycarbonyl)pentyl] indocarbocyanine iodide (IC3-OSu), and *N*-ethyl-*N*′-[5-(*N*″-succinimidyloxycarbonyl)pentyl]-3,3,3',3'-tetramethyl-2,2'-indodicarbocyanine iodide (IC5-OSu) were purchased from Dojindo Molecular Technologies, Inc. (Kumamoto, Japan). An XL-TrypKit was purchased from APRO Science Inc. (Tokushima, Japan). A ProteoExtract™ Protein Precipitation Kit was purchased from Merck Millipore (Darmstadt, Germany). An anti-FLAG monoclonal antibody, CCCP and L-NMMA were obtained from Sigma-Aldrich (St. Louis, MO). An anti-Tom20 antibody was purchased from Santa Cruz (Santa Cruz, CA). The anti-HSP60 antibody was purchased from BD Transduction Laboratories (San Jose, CA). Rotenone was obtained from Nacalai Tesque (Kyoto, Japan).

Two-dimensional fluorescence difference gel electrophoresis (2D-DIGE)

After measuring the weight of the mouse brain samples, we added ten volumes of lysis buffer (10 mM Tris (pH 8.0), 7 M urea, 2 M thiourea, 5 mM magnesium acetate, 4% (w/v) CHAPS, 4 mM protease inhibitor) and homogenized and sonicated the samples. After centrifugation at 8000 × g for 10 min, the supernatant was used for 2D-DIGE analyses.

Proteomic comparison of brain tissues between three pairs of wild-type and DJ-1$^{-/-}$ mice was performed by 2D-DIGE analyses using a pooled sample, the equivalent mixture of all samples, as the internal standard. The samples were labeled using 400 pmol of fluorescent dye reagents (IC3-OSu or IC5-OSu) per 50 µg of protein extract. Individual samples and the internal standard were labeled with IC5 and IC3, respectively. The labeling reaction was performed at 37 ºC for 20 min and quenched by the addition of 1 mM ethanolamine at room temperature (RT) for 10 min in the dark.

The internal standard IC3-labeled samples and the individual IC5-labeled samples were pooled equivalently, followed by precipitation using the ProteoExtract™ Protein Precipitation Kit to clean up the samples and to exchange the buffer for isoelectric focusing (IEF) buffer (5 M urea, 2 M thiourea, 2% CHAPS, 3% SB3-10, 1% DTT, and 2% Pharmalyte 3-10). Samples containing 15 µg of protein each were loaded in analytical gels, and samples containing 200 µg of protein each were loaded in preparative gels for protein identification. 2D-PAGE was conducted using immobilized dry strip gels with a pH range of 4-7, and SDS-PAGE (%T = 7.5 and %C = 3.0) was performed as described previously with slight modifications[S1] on duplicates of each individual sample.

After 2D-PAGE, gels were scanned on a Typhoon FLA 9500 laser scanner (GE Healthcare), and IC3- and IC5-labeled protein spots were detected at excitation/emission wavelengths of 550/570 nm and 649/670 nm, respectively. Duplicate images of each individual sample and the standard sample were uploaded into Progenesis SameSpots gel image analysis software ver. 4.5 (Totallab, Newcastle, UK), and automated inter-gel alignment, spot detection, normalization, and comparative determination were performed. The computer-detected spots were listed by fold change and statistical significance for the comparison of the normalized spot volumes between the wild-type and knockout groups. We considered a spot to be significantly altered between the groups when the fold change was greater than 1.5 or if $p < 0.05$ by ANOVA.

Spot picking and in-gel trypsin digestion

Differentiated protein spots were mechanically picked from 2 preparative gels using Ettan Spot Picker (GE Healthcare). In-gel digestion was performed on the picked gel spots using an XL-TrypKit according to the manufacturer’s instructions,

and the tryptic peptides from each spot were subjected to liquid chromatography with matrix-assisted laser desorption ionization (LC-MALDI) analysis and protein identification.

LC-MALDI analysis

LC-MALDI analyses were carried out using the DiNa-MaP direct nanoLC and MALDI fraction system (KYA Technologies, Tokyo, Japan) and a MALDI-TOF/TOF 5800 system (SCIEX, Framingham, MA, USA). Samples were fractionated into 79 fractions using DiNa-MaP. The mobile phase was 2% MeCN containing 0.1% TFA (solvent A) and 70% MeCN containing 0.1% TFA (solvent B). The matrix solution was prepared with a concentration of 4 mg/mL CHCA and 0.08 mg/mL ammonium citrate in solvent B and pumped at a flow rate of 1400 nL/min. After the sample was loaded on a trap column (HiQ sil C18W-3, 0.5 × 1 mm, KYA Technologies), the valve was switched, and peptides were separated with an analytical column (HiQ sil C18W-3, 0.1 × 50 mm, KYA Technologies) at a flow rate of 300 nL/min. The LC gradient was as follows: 0–2 min, 0–5% solvent B; 2–25 min, 5–50% solvent B; 25–30 min, 50–100% solvent B; 30–40 min, 100–0% solvent B; 40–55 min, 0% solvent B. Mass spectra were acquired from *m/z* 800–4000 using a TOF/TOF™ 5800 system operated with TOF/TOF™ Series Explorer™ software version 4.1 (SCIEX), and each spectrum was obtained by accumulating 800 laser shots. Bradykinin fragment 2–9 (*m/z* 904.47), angiotensin I (*m/z* 1296.69), Glu^1-fibrinopeptide B (*m/z* 1570.68), ACTH fragment 1-17 (*m/z* 2093.09), ACTH fragment 18-39 (*m/z* 2465.20), and ACTH fragment 7–38 (*m/z* 3657.93) were used for external calibration. MS/MS spectra were automatically acquired from the intensive precursor ions (S/N > 50).

Protein identification

Protein identification was performed via an MS/MS ion search using ProteinPilot™ software (ver.4.5; SCIEX). The search parameters were as follows: database, uniprot_sprot_can+iso_20100622; species, mouse; Cys alkylation, iodoacetamide; digestion, trypsin; and special factors, Gel-based-ID and max missed cleavage, 1. Protein identifications were considered to be correct based on the following selection criteria: proteins with a protein score (ProtScore) of > 1.3

(unused, $p < 0.05$, 95% confidence) and proteins having at least 2 peptides with an ion score above the 95% confidence threshold.

Gene ontology (GO) analysis

Differentially expressed proteins between WT and KO were further analyzed for gene ontology analysis using the PANTHER classification system (http://www.pantherdb.org).[S2]

Pathway analysis

To extract molecular networks biologically relevant to differentially expressed proteins, pathway analysis was performed using KeyMolnet ver. 6.2 (KM Data, Tokyo, Japan).[S3] The "Protein ID" list of differentially expressed proteins was uploaded into KeyMolnet, and the "Interaction search" algorithm was used to generate the network of molecular interactions in two paths from the starting points, including direct activation/inactivation, transcriptional activation/repression, and complex formation. The canonical pathways associated with differentially expressed proteins in the brain tissues of DJ-1$^{-/-}$ mice were extracted from the KeyMolnet knowledge base.

Another pathway analysis was performed using the Kyoto Encyclopedia of Genes and Genomes (KEGG) (https://www.genome.jp/kegg/pathway.html), which is a publicly accessible knowledge base that covers a wide range of pathway maps on metabolic, genetic, environmental, and cellular processes, and human diseases[S4]. By uploading the list of "Protein ID" of differentially expressed proteins, KEGG extracts relevant pathways composed of the proteins enriched in the given list, followed by statistical evaluation.

Confocal microscopy

Cells were fixed with 4% paraformaldehyde in PBS and permeabilized with 0.2% Triton X-100 in PBS. Cells stained with the appropriate antibodies were imaged using a laser scanning microscope (FV1000, Olympus, Tokyo, Japan) fitted with a UPlanSApo 40×/0.9NA lens (Olympus, Tokyo, Japan). To quantify the compaction index of mitochondria,

midplane images of cells immunostained for Tom20 were obtained as described previously[S5]. In ImageJ (NIH), data in the Tom20 channel were converted into binary data, and the area and perimeter of mitochondria within the cell of interest (selected using the "region of interest" tool) were measured using the "analyze particles" function. The compaction index (which is the perimeter of a circle with the same area as the object of interest divided by the actual perimeter of the object of interest) was calculated from the perimeter (P) and the area (A) using the following formula: $(2\pi \times ((A/\pi)^{1/2}))/P$.

Construction of Plasmids

To construct targeting vectors, fragments were amplified from genomic DNA purified from SH-SY5Y cells by Polymerase chain reaction (PCR) and inserted into pMulti-ND 1.0 vector (a kind gift from Dr. J. Takeda and K. Horie at Osaka University and Nara Medical University)[S6]. Construct for ingle cysteine mutant of DJ-1 were generated using the PrimeSTAR Mutagenesis Basal Kit (Takara, Otsu, Shiga, Japan) and appropriate primers following manufacturer's instructions. TALEN plasmids were constructed as described previously[S7].

Establishment of cell lines

SH-SY5Y cells were transfected with targeting vector and TALEN plasmids as described in the Materials and Methods. Selection and maintenance of transfectants were performed in the presence of hygromycin and/or puromycin for DJ-1$^{-/-}$ cells and zeocin and/or G418 for Parkin^{C323S} cells. Colonies were resuspended and grown in 96-well plates at a density of about one cell per well. Genotyping of each cell line was performed by PCR and Southern blotting of genomic DNA. After genotyping, cells were transfected with plasmids expressing cre recombinase to remove the selection marker, and then were resuspended in 96-well plates at a density of about 1/10 cell per well to be cloned.

Southern blot analysis

Southern blot analysis was performed using the DIG system (Merck KGaA, Darmstadt, Germany) according to the manual.

Flow cytometry

Cells were incubated with Tetramethylrhodamine, methyl ester (TMRM, abcam, Cambridge, UK), JC-1 (Dojindo Molecular Technologies, Japan) or MitoSOX (Life Technologies, CA) for 30 min at 37°C under regular cell culture conditions. Then, they were washed 3 times in warm PBS, detached with trypsin, washed and resuspended in PBS and sorted using a FACScan™ (BD Biotechnology).

Immunoblot analysis

Immunoblotting was performed as described previously[S8]. Briefly, total protein was separated by SDS-PAGE and transferred to a polyvinylidene difluoride (PVDF) membrane. The membrane was first blocked in Tris-buffered saline containing 0.05% Tween 20 (TBS-T) and 5% nonfat skim milk (Nacalai, Japan) and was then incubated with the primary antibody. After washing with TBS-T, the membrane was incubated with the secondary antibody in TBS-T containing nonfat dry milk. After washing, the blots were developed with ECL Plus Western blotting substrate (Buckinghamshire, UK). Band intensities on the immunoblots were quantified by densitometry using NIH ImageJ software. The uncropped images of the immunoblots are shown in Supplemental Fig. S8.

Detection of S-nitrosylated proteins by SNO-RAC

S-nitrosylated proteins were detected by using the SNO-RAC method as described[S9], with some modifications[S8]. Briefly, SNO-RAC resins were prepared as described. A total of 250 μL of cell lysates was diluted with 750 μL of HEN buffer (250 mM HEPES, 1 mM EDTA, 0.1 mM neocuproine (pH 8.0)) and incubated with 1% SDS (final concentration) and 0.1% methyl methanethiosulfonate (Sigma-Aldrich) at 50°C for 25 min. Proteins were precipitated with acetone, washed three times with 70% acetone and resuspended in 200 μL of HENS buffer (HEN containing 1% SDS). This suspension was added to 50 μL of resin slurry in the presence of sodium ascorbate (Fluka, final 20 mM) and mixed by rotation in the dark for 3 h, and the resin was then washed with 4 × 1 mL HENS buffer. The captured proteins were eluted with 30 μL of

HENS buffer containing 100 mM 2-mercaptoethanol for 20 min at RT, and 20 μL of each eluent was used for SDS-PAGE analysis.

Supplemental References

S1. Miura, Y. ***et al.*** Age-dependent variations of cell response to oxidative stress: proteomic approach to protein expression and phosphorylation. *Electrophoresis.* **26**, 2786-96 (2005)

S2. Mi, H., Muruganujan, A., Casagrande, J.T., & Thomas P.D. Large-scale gene function analysis with the PANTHER classification system. *Nat. Protoc.* **8**, 1551-66 (2013)

S3. Hirayama, M. ***et al.*** Integrated proteomics identified novel activation of dynein IC2-GR-COX-1 signaling in neurofibromatosis type I (NF1) disease model cells. *Mol. Cell. Proteomics.*

S4. Ogata, H. ***et al.*** KEGG: Kyoto Encyclopedia of Genes and Genomes. *Nucleic Acids Res.* **27**, 29-34 (1999)

S5. Narendra, D., Kane, L.A., Hauser, D.N., Fearnley, I.M., & Youle, R.J. p62/SQSTM1 is required for Parkin-induced mitochondrial clustering but not mitophagy; VDAC1 is dispensable for both. *Autophagy.* **6**, 1090-106 (2010).

S6. Inoue, N., Ikawa, M., Isotani, A. & Okabe, M. The immunoglobulin superfamily protein Izumo is required for sperm to fuse with eggs. *Nature.* **434**, 234–238 (2005)

S7. Sakuma, T. et al. Repeating pattern of non-RVD variations in DNA-binding modules enhances TALEN activity. Sci. Rep. 3, 3379 (2013)

S8. Ozawa, K. ***et al.*** S-nitrosylation regulates mitochondrial quality control via activation of parkin. *Sci. Rep.* **3**, 2202 (2013)

S9. Forrester, M.T. ***et al.*** Proteomic analysis of S-nitrosylation and denitrosylation by resin-assisted capture. *Nat. Biotechnol.* **27**, 557-9 (2009).
